# Acetate modulates host physiology and bacterial energetics to improve antibiotic efficacy during obesity-associated infection

**DOI:** 10.1128/mbio.01630-26

**Published:** 2026-10-16

**Authors:** Arsalan Hussain, Prateek Bhardwaj, Ashish Kumar Ray, Aakash Singh, Abhiroop Sett, Ranjana Pathania

**Affiliations:** 1 Department of Biosciences and Bioengineering, Indian Institute of Technology Roorkee 729296, Roorkee, India; Universiteit Gent, Gent, Belgium

**Keywords:** aminoglycosides, gram-negative infections, host-directed therapy, short-chain fatty acids, treatment failure

## Abstract

Antibiotic efficacy is conventionally attributed to the susceptibility of the infecting pathogen. However, obese patients experience disproportionately poor infection outcomes despite receiving antibiotics to which pathogens remain genetically susceptible. These observations suggest that host-associated factors can influence treatment outcomes independently of the bacterial genotype, although the underlying mechanisms remain poorly understood. Using a mechanistic comparison of diet-induced obese and healthy mice infected with *Acinetobacter baumannii*, we demonstrate that the effect of antibiotics is not solely a property of the pathogen but can be governed by a gut microbiota-derived metabolite acting on both host immunity and bacterial physiology. We identify obesity-associated acetate depletion as a key driver of antibiotic treatment failure, linking gut microbial metabolism to antibiotic activity during infection. We observed that this depletion impaired innate immune activation, reducing cytokine responses and neutrophil-mediated bacterial clearance. Under these conditions, *A. baumannii* displayed enhanced tolerance to bactericidal antibiotics, particularly tobramycin. Given these findings, we investigated whether acetate could be leveraged therapeutically to restore antibiotic efficacy. We observed that oral acetate supplementation remodeled the dysbiotic microbiome, whereas systemic acetate restored innate immune competence and neutrophil-mediated bacterial clearance. Most importantly, acetate reversed antibiotic tolerance and restored aminoglycoside killing by directly sensitizing host-induced tolerant bacterial populations. Mechanistically, acetate directly reprogrammed bacterial physiology to enhance aminoglycoside-mediated killing. Combined acetate-tobramycin treatment resulted in eradication of tolerant populations and markedly improved bacterial clearance *in vivo*. Our findings establish a mechanistic link between host metabolic state and antibiotic efficacy and identify acetate as a therapeutic intervention for obesity-associated gram-negative infections.

**IMPORTANCE:**

Antibiotic treatment failure in obese patients remains poorly understood and therapeutically unaddressed. This study reveals that host metabolic state can control antibiotic response through a gut microbiota-derived metabolite that acts on both host defense and bacterial physiology. We identify obesity-driven depletion of microbiota-derived acetate as a previously unrecognized determinant of antibiotic failure, operating independently of bacterial resistance. By simultaneously restoring gut microbial balance, rescuing impaired innate immunity, and directly sensitizing pathogens to aminoglycoside killing through bacterial membrane alteration, acetate supplementation addresses the host-pathogen-microbiome axis through a single intervention. Given its established clinical safety, acetate represents a compelling combination therapy alongside aminoglycosides in obesity-associated gram-negative infections in a population for which host-directed treatment strategies are currently limited.

## INTRODUCTION

Bacterial infections remain a leading cause of morbidity and mortality worldwide, and their management is increasingly complicated by therapeutic failure even in the absence of classical genetic resistance (1, 2). Conventional antimicrobial susceptibility frameworks classify pathogens as susceptible or resistant based on heritable determinants, including acquisition of resistance genes (3), enzymatic drug inactivation (4), and modification of antibiotic targets (5). Yet these frameworks frequently fail to explain treatment failure during infections caused by pathogens that remain genetically susceptible to the prescribed antibiotic (6, 7). Such discrepancies point to an increasingly recognized phenomenon: antibiotic tolerance, wherein pathogens survive bactericidal antibiotic exposure through transient physiological adaptation rather than stable genetic resistance (8, 9).

Unlike resistance, antibiotic tolerance is highly context-dependent (10, 11). Bacterial susceptibility to antibiotics is shaped not only by intrinsic physiology but also by the environment encountered during infection (12, 13). Increasing evidence suggests that antibiotic efficacy *in vivo* emerges from interactions between bacterial physiology and host immunity, with immune-mediated pathogen clearance often amplifying antibiotic activity through Toll-like receptor (TLR)-dependent pathways (14, 15). Normal host immune function can, therefore, enhance antibiotic efficacy, whereas physiological disruption may fundamentally alter treatment outcomes (15).

In this context, obesity represents a clinically relevant state of metabolic and immune dysregulation with the potential to alter antibiotic efficacy (16). Obesity is associated with disproportionately high rates of nosocomial infection, sepsis-related hospitalization, and infection-associated mortality (17–19). Clinically, obese individuals frequently exhibit poor antibiotic response despite adequate drug exposure and the absence of overt resistance mechanisms, suggesting that obesity reshapes the host environment in ways that impair treatment outcomes independently of pathogen genotype (20). What distinguishes the obese host immunologically is not merely increased inflammation but profound inflammatory dysregulation (21). This is caused by a state of chronic metabolic inflammation that exhausts rather than primes acute innate responses.

Here, we investigated how obesity-associated immune and microbiome dysfunction impairs antibiotic efficacy during gram-negative infection. We used a murine model of diet-induced obesity and systemic *A. baumannii* infection, which is a WHO-designated critical-priority pathogen and leading cause of hospital-acquired infection (22). Studies have shown that diet-induced obesity disrupts the gut microbiome, depleting microbial taxa responsible for producing short-chain fatty acids (SCFAs) (23, 24), of which acetate is the most abundant in systemic circulation and a key regulator of innate immune function (25). However, the role of obesity-associated acetate depletion to cause antibiotic treatment failure still remains unexplored. Here, we provide mechanistic evidence where such a depletion represents a previously unrecognized determinant of treatment failure, operating at the intersection of gut microbiome composition, host innate immunity, and bacterial membrane energetics, and propose exogenous acetate supplementation as a standalone immune-restorative intervention or in combination with tobramycin as a tractable strategy to improve treatment outcomes in obese hosts.

## RESULTS

### Diet-induced obesity increases susceptibility to infection

**Fig 1:**
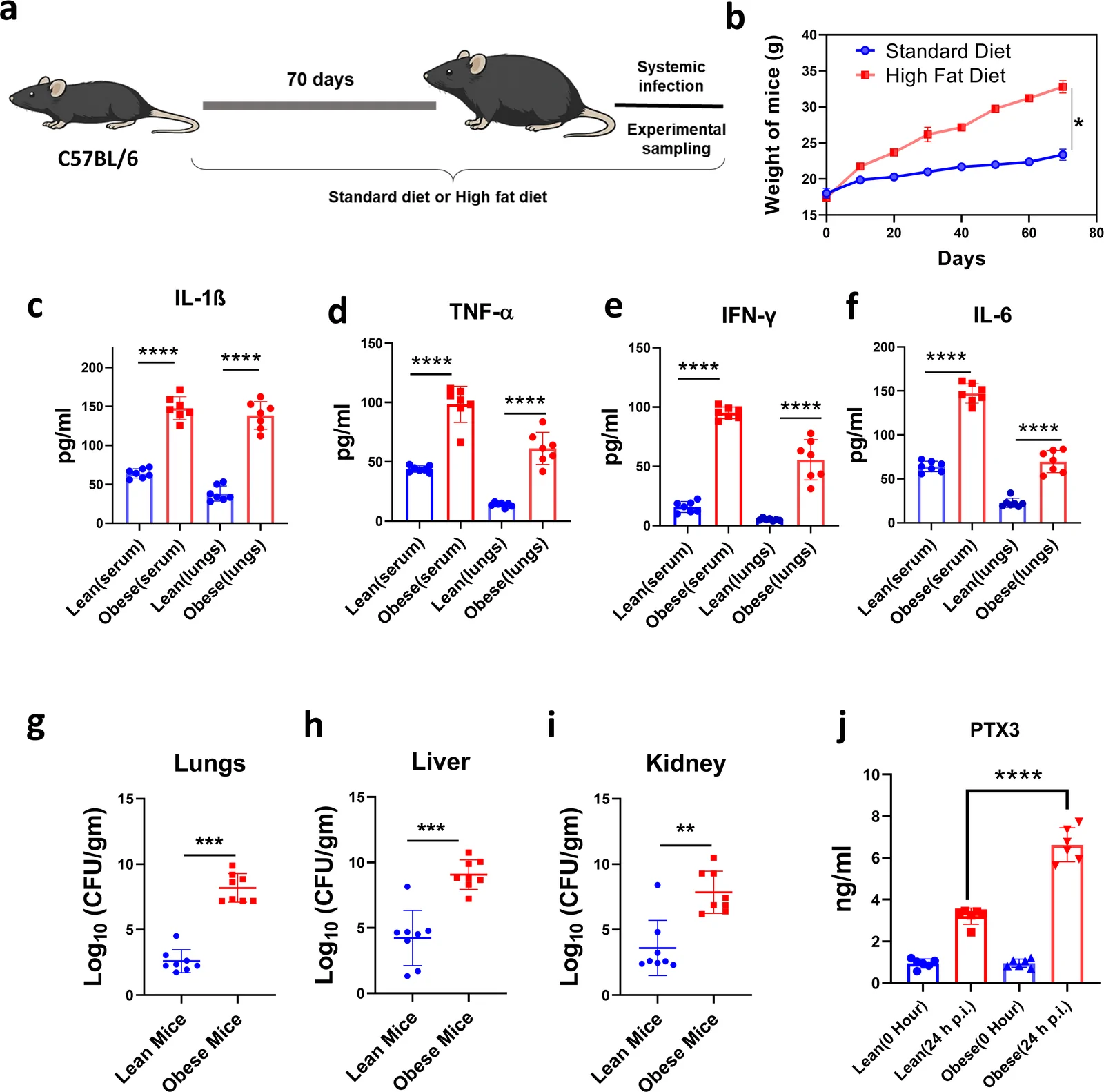
High-fat diet enhances systemic inflammation and bacterial burden in a murine infection model. (**a**) Schematic representation of the experimental design. C57BL/6 mice were maintained on either a standard diet or HFD for 70 days, followed by systemic infection and subsequent sample collection. (**b**) Longitudinal body weight measurements of mice fed a standard diet or HFD over 70 days. Body weight was recorded at regular intervals throughout the feeding period. Data represent mean ± s.d.; *n* = 10 biologically independent animals per group. Statistical significance was determined using an unpaired two-tailed Student’s *t*-test. (**c–f**) Quantification of cytokines in serum and lungs from lean and obese mice at uninfected baseline. Levels of (**c**) IL-1β, (**d**) TNF-α, (**e**) IFN-γ, and (**f**) IL-6 were measured using enzyme-linked immunosorbent assay (ELISA) according to the manufacturer’s instructions. Cytokine concentrations are expressed as pg/mL. Data are shown as mean ± s.d.; *n* = 7 animals per group. *P* values were calculated using one-way analysis of variance (ANOVA); Tukey’s multiple comparisons test. (**g–i**) Bacterial burden in (**g**) lungs, (**h**) liver, and (**i**) kidneys of lean and obese mice following infection with *A. baumannii* 17978 at 10^7^ colony-forming unit (CFU)/mL. Organs were aseptically harvested, homogenized in sterile phosphate-buffered saline, serially diluted, and plated for colony-forming unit (c.f.u.) enumeration. Bacterial loads are presented as log_10_ c.f.u. per gram of tissue. Data are shown as mean ± s.d.; *n* = 8 animals per group. Statistical analysis was performed using the two-tailed Mann-Whitney *U* test (nonparametric test) for comparisons between lean and obese mice. (**j**) Serum levels of pentraxin-3 (PTX3) measured at baseline (0 h) and 24 h post-infection in lean and obese mice using ELISA. Data are presented as ng/mL. Each dot represents an individual animal. Significance determined by one-way ANOVA followed by Tukey’s multiple comparison test (*P < 0.01; **P < 0.001; ***P < 0.003; ****P < 0.0001; ns, nonsignificant).

To investigate how obesity-associated immune remodeling shapes susceptibility to opportunistic pathogens, we established a diet-induced obesity model in C57BL/6 mice and characterized its metabolic, immunological, and infection-associated features (Fig. 1a). C57BL/6 mice fed a high-fat diet (HFD) developed an obese phenotype, exhibiting an average ~10 g increase compared with normal-diet (ND) controls (Fig. 1b). This was accompanied by significant elevations in total cholesterol and triglyceride levels in both serum samples and liver homogenates (Fig. S1a and b).

Obesity is characterized by chronic low-grade inflammation that disrupts immune homeostasis (26). Consistent with this, obese mice exhibited significantly elevated basal levels of IL-1β, IL-6, TNF-α, and IFN-γ in both serum and lung tissues compared with lean controls (Fig. 1c through f). These cytokines are key mediators of innate and adaptive immune activation, which co-ordinates proinflammatory signaling, leukocyte recruitment, and antimicrobial effector functions (27). Their persistent elevation reflects a state of unresolved systemic inflammation. Such an increase across systemic and lung homogenates indicates that obesity-associated inflammation extends beyond the circulation into peripheral tissues that serve as primary sites of bacterial infection.

To determine the functional consequences of such alterations, lean and obese mice were systemically challenged with *A. baumannii* ATCC 17978 (28). Despite the heightened baseline inflammation, obese mice exhibited a marked defect in bacterial clearance, with significantly higher bacterial burdens in the lungs, liver, and kidneys compared with lean controls (Fig. 1g through i). Histological examination of lung sections revealed clear differences in infection-associated pathology between lean and obese mice (Fig. S1c through f). Lean mice displayed normal alveolar architecture with well-defined air spaces and minimal cellular infiltration (Fig. S1c), indicating healthy lung tissue. Lean mice with infection showed moderate inflammatory cell infiltration and mild thickening of alveolar septa, suggesting an active but controlled immune response to infection (Fig. S1d). Obese mice exhibited mild structural alterations and slight inflammatory infiltration compared with lean controls, indicating a pre-existing low-grade inflammatory state (Fig. S1e). In contrast, obese mice with infected lungs demonstrated extensive inflammatory cell accumulation, alveolar septal thickening, and partial consolidation of air spaces, reflecting exacerbated pulmonary pathology and increased susceptibility to infection in the obese host (Fig. S1f).

To evaluate whether this impaired bacterial clearance was accompanied by systemic inflammatory stress, we measured circulating PTX3, which is an acute-phase protein rapidly induced during microbial invasion and a biomarker of infection severity (29). PTX3 levels were minimal at baseline in both groups; however, at 24 h post-infection, obese mice exhibited a pronounced increase in serum PTX3 compared with lean controls (Fig. 1j). This elevation indicates extensive tissue damage.

### Obesity leads to impaired cytokine kinetics and innate immune activation following infection

Effective immunity against bacterial pathogens depends not only on the magnitude of inflammation but also on its precise temporal regulation, which is characterized by rapid inflammatory induction followed by timely resolution (30, 31). To assess how obesity influences this temporal coordination during infection, we longitudinally profiled systemic inflammatory responses in lean and obese mice (Fig. S2a). Serum samples were collected prior to infection and at 1, 7, and 14 days post-infection with *A. baumannii*. In lean mice, *A. baumannii* infection elicited a coordinated early inflammatory response. At day 1 post-infection, serum levels of TNF-α, IFN-γ, IL-1β, and IL-6 were significantly elevated relative to pre-infection baseline, consistent with effective early innate immune activation (Fig. S2b and e). This response was transient, with cytokine levels progressively declining by day 7 and returning close to baseline by day 14, reflecting proper termination of the inflammatory response. In contrast, obese mice exhibited an altered cytokine profile. Although baseline cytokine levels were elevated prior to infection, consistent with obesity-associated metaflammation, obese mice failed to elicit a comparable early cytokine surge following *A. baumannii* challenge. At day 1 post-infection, serum TNF-α, IFN-γ, IL-1β, and IL-6 levels were significantly lower than those observed in lean mice, indicating a disorganized acute inflammatory response (Fig. S2b through e). Notably, this early impairment was followed by a sustained elevation of proinflammatory mediators, with cytokine levels remaining significantly higher in obese mice than in lean controls through day 14 post-infection (Fig. S2b through e). This aberrant temporal pattern suggests defective regulation of the immune response, which may contribute to impaired bacterial clearance.

To assess the outcome of immune response at the site of infection, as outlined in the experimental workflow (Fig. S3a), lean and diet-induced obese C57BL/6 mice were infected with *A. baumannii*, and organ samples were collected at baseline (day 0) and at day 1 post-infection for transcriptional analysis. To address this, we quantified *Tlr4* expression (32) together with downstream proinflammatory cytokines and neutrophil-recruiting chemokines in both lung and kidney homogenates following infection, normalizing the expression to lean mice at baseline (day 0). At day 1 post-infection, *Tlr4* transcripts were strongly induced in lean mice (lung ~4.6-fold; kidney ~5.9-fold). However, this induction was significantly attenuated in obese mice (lung ~3.2-fold; kidney ~2.8-fold), corresponding to approximately 1.4-fold and 2-fold lower expression compared with lean controls, respectively (Fig. S3b). This attenuation indicates an early defect in pathogen sensing in lung and kidney homogenates.

Consistent with the differential *Tlr4* response at the transcript level, obese mice displayed reduced cytokine induction, which is the endpoint of downstream TLR4 signaling. In the lung homogenates, lean mice exhibited strong upregulation in transcript levels of *Il*-*6* (~10-fold), *Ifn-*γ (~16-fold to 17-fold), *Tnf-*α (~15-fold), and *Il1*β (~8-fold), whereas obese mice showed significantly lower comparative induction (*Il6* ~7-fold*; Ifn*γ ~7-fold to 8-fold; *Tnf*α ~11-fold; and *Il1*β, ~5-fold) (Fig. S3c through f). A similar pattern was observed in the kidney, where lean mice demonstrated significant induction (*Il-6* ~12-fold; *Ifnγ* ~16-fold; *Tnf*α ~12-fold to 13-fold; *Il1*β ~ 4-fold), while obese mice exhibited attenuated comparative response (*Il-6* ~6-fold to 7-fold; *Ifn*γ ~7-fold; *Tnf*α ~9-fold; *Il1*β ~ 2-fold) (Fig. S3c through f).

This reduced cytokine amplification was accompanied by diminished chemokine expression. In the lung, *Cxcl2* and *Cxcl3* (neutrophil chemoattractant) transcripts were strongly upregulated in lean mice (~14-fold and ~12-fold) but significantly lower in obese mice (~10-fold; ~8-fold respectively; Fig. S3g through h). Similarly, in the kidney, lean mice showed marked upregulation (*Cxcl2* ~12-fold; *Cxcl3* ~15-fold), whereas obese mice displayed reduced induction (*Cxcl2* ~9-fold to 10-fold; *Cxcl3* ~8-fold to 9-fold; Supplementary Fig g-h). This suggests impaired neutrophil recruitment at the site of infection in obese mice. Given that neutrophil recruitment is tightly regulated by SCFA signaling through free fatty acid receptors present on them, we next examined the expression of *Ffar2* and *Ffar3*. Notably, infection-induced upregulation of *Ffar2* was significantly attenuated in obese mice compared with lean controls (lung ~1.9-fold vs ~3.6-fold; kidney ~1.8-fold vs ~3.4-fold; Fig. S3i). A similar pattern was observed for *Ffar3*, which was extensively induced in lean mice (lung ~3.8-fold; kidney ~4.3-fold) but markedly reduced in obese mice (lung ~2.2-fold; kidney ~2.0-fold; Fig. S3j).

**Fig 2:**
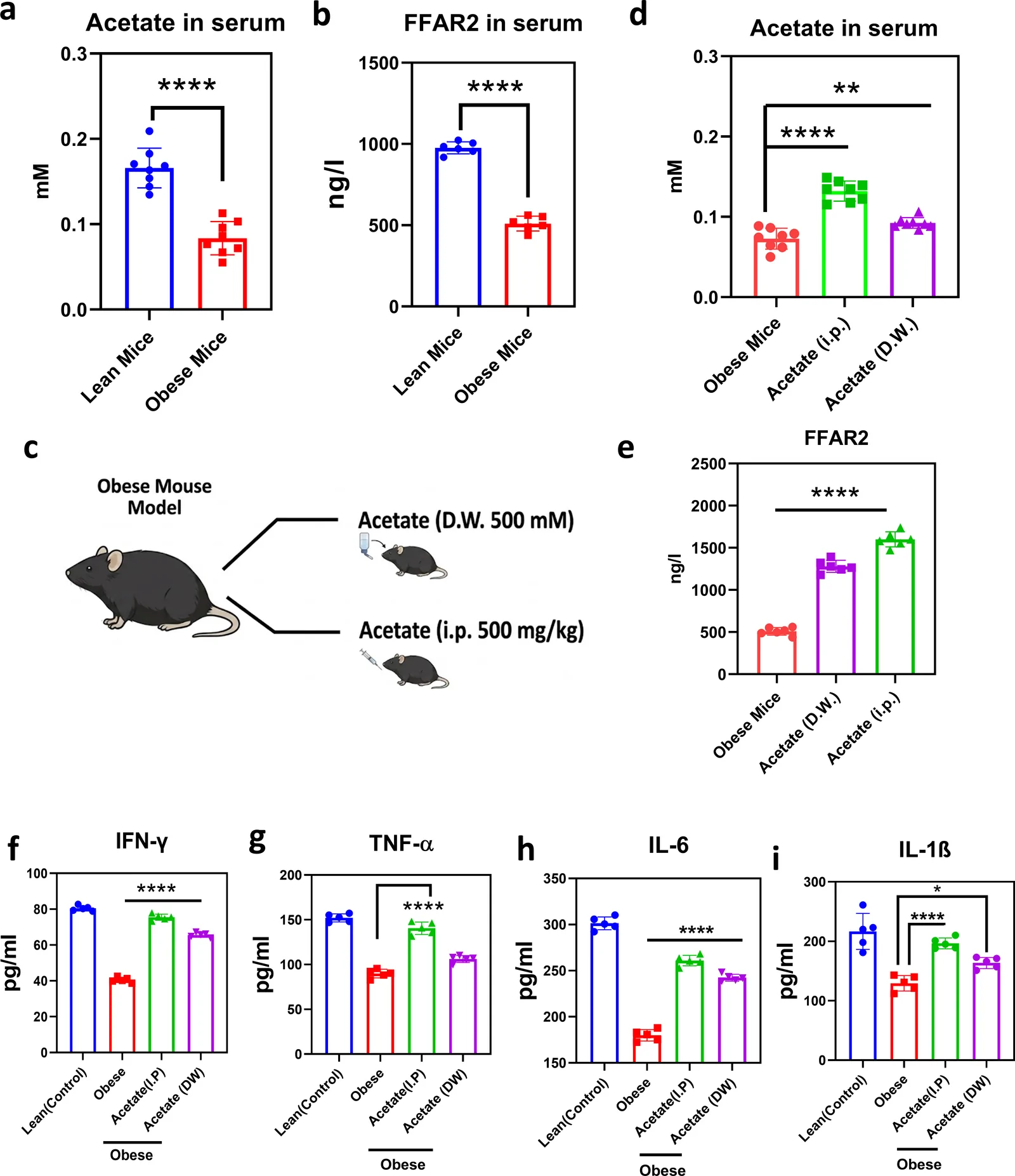
Acetate supplementation restores immune response in obese mice following infection. (**a**) Serum acetate levels in lean and obese mice measured using a colorimetric assay kit according to the manufacturer’s instructions. Data are presented in mM. (**b**) FFAR2 levels in serum from lean and obese mice quantified by ELISA. (**c**) Schematic representation of acetate administration in obese C57BL/6 mice. Acetate was delivered either via drinking water (DW; 500 mM) or intraperitoneal (i.p. 500 mg/kg body weight) injection prior to infection. (**d**) Serum acetate concentrations in obese mice following different modes of acetate delivery (i.p. and drinking water), measured 24 h post-treatment using a colorimetric assay (*n* = 8 mice per group). (**e**) Serum FFAR2 levels in obese mice following acetate supplementation (DW or i.p.), quantified by ELISA at 24 h post-treatment (*n* = 6 mice per group). (**f–h**) Circulating cytokine levels measured following infection in lean control and obese mice with or without acetate supplementation. Serum levels of (**f**) IFN-γ, (**g**) TNF-α, (**h**) IL-6, and (**i**) IL-1β were quantified by ELISA. Blood samples were collected 24 h post-infection (*n* = 5 mice per group). Data are shown as mean ± s.d. Cytokine concentrations are expressed as pg/mL. Significance determined by one-way ANOVA followed by Tukey’s multiple comparison test (*P < 0.01; **P < 0.001; ***P < 0.003; ****P < 0.0001; ns, nonsignificant).

The attenuated induction of *Ffar2* and *Ffar3* transcripts in lung and kidney tissues of obese mice suggested impaired SCFA-mediated immune signaling post-infection. As acetate constitutes approximately 60% of circulating SCFAs and is the most abundant and consistently detectable SCFA in serum (33), we next examined whether reduced receptor response was associated with diminished ligand availability. We measured circulating acetate levels, which were significantly reduced in obese mice at baseline compared with lean controls (Fig. 2a). To determine whether reduced SCFA sensing was also reflected at the receptor level, we quantified circulating FFAR2 protein levels at baseline. Obese mice exhibited significantly lower serum FFAR2 levels compared with lean controls (Fig. 2b), indicating that suppression of this key SCFA receptor is an intrinsic feature of the obese host, independent of the infection status.

### Gut microbiome dysbiosis in obesity reduces SCFA availability and antibiotic tolerance in *A. baumannii*

The downregulation of *Ffar2* and *Ffar3* expression observed in obese mice during *A. baumannii* infection suggested a defect in SCFA-mediated immune signaling. Given that acetate is predominantly generated through microbial fermentation of dietary substrates in the gut (34), we next hypothesized that reduced systemic acetate levels in HFD-induced obese mice may reflect alterations in gut microbial community structure. We therefore assessed whether obesity-associated acetate deficiency is linked to compositional and functional changes in the gut microbiome. To address this, we performed 16S profiling of fecal samples collected from lean and obese C57BL/6 mice. Community diversity analysis revealed a significant reduction in microbial richness and evenness in obese mice, as evidenced by decreased Shannon index, reduced Chao1 richness, and lower Simpson diversity, indicating loss of microbial complexity under HFD conditions (Fig. S4a through c).

Phylum-level analysis demonstrated a marked reduction in Bacteroidota, a major SCFA-producing phylum, accompanied by an increased relative abundance of Bacillota (Firmicutes) in obese mice (Fig. S4d). This shift results in an elevated Firmicutes-to-Bacteroidota ratio, which is consistently associated with obesity and enhanced energy harvest from the diet, low-grade inflammation, and metabolic dysfunction (35). Bacteroidota are one of the principal gut producers of SCFAs through carbohydrate fermentation pathways (36). The functional consequence of Bacteroidota depletion for circulating acetate is amplified by its unique disposition. Here, unlike butyrate, which is largely consumed by colonocytes (37), and propionate, which is extracted by the liver (38), acetate escapes first-pass metabolism to become the dominant SCFA in peripheral blood (39).

Given that HFD-induced obesity resulted in marked gut microbial dysbiosis and depletion of SCFA-producing taxa, we next asked whether these microbiome alterations influence antibiotic efficacy during infection. Emerging evidence suggests that host metabolic state and microbiota-derived metabolites can shape bacterial physiology *in vivo*, thereby modulating susceptibility to antimicrobial agents independent of classical resistance mechanisms (10).

To test this hypothesis, lean and diet-induced obese mice infected with *A. baumannii* were treated with clinically relevant antibiotics, including tobramycin, polymyxin B, meropenem, and rifampicin (Fig. S5a). Despite the administration of body weight-adjusted antibiotic doses, obese mice exhibited markedly reduced antibiotic-mediated bacterial clearance across multiple organs compared with lean mice. In the lungs, tobramycin-mediated killing was substantially diminished in obese animals, and similar reductions in efficacy were observed for polymyxin B, meropenem, and rifampicin (Fig. S5b). A comparable trend was observed in the liver, where tobramycin activity was significantly impaired in obese mice and rifampicin also showed reduced efficacy, whereas polymyxin B and meropenem displayed no statistically significant differences between groups (Fig. S5c and d). Strikingly, in the kidney, reduced antibiotic activity in obese mice was observed across all tested antibiotics, including tobramycin, polymyxin B, meropenem, and rifampicin (Fig. S5d). These results demonstrate reduced bactericidal efficacy of multiple antibiotics in obese hosts across different organs, suggesting that host-associated factors may contribute to impaired antibiotic response.

Among the antibiotics tested, tobramycin displayed one of the most pronounced losses in efficacy in obese mice, particularly in pulmonary and renal tissues. Since aminoglycoside uptake depends on bacterial membrane potential and metabolic activity (40), these findings are consistent with the possibility that altered host metabolic cues influence antibiotic susceptibility during infection. Notably, our earlier observations indicating depletion of acetate, a key microbiota-derived metabolic substrate, in obese hosts further support a model in which host metabolic alterations indirectly impair aminoglycoside activity *in vivo*. Moreover, to exclude the possibility that reduced efficacy resulted from altered drug exposure, we next assessed systemic levels of tobramycin following intraperitoneal administration. Measurement of serum drug concentrations 30 min post-injection (2) revealed no significant difference between lean and obese mice (Fig. S5e), indicating that pharmacokinetic differences are unlikely to account for the observed reduction in antibacterial activity. Together, these findings suggest that obesity-associated metabolic dysregulation alters the infection microenvironment in a manner that diminishes antibiotic-mediated bacterial clearance across multiple tissues.

### Exogenous acetate supplementation restores immune signaling and reduces bacterial burden in obese mice

Acetate has been reported to regulate neutrophil recruitment and inflammatory responses through FFAR2-dependent signaling (25). Based on this, we hypothesized that restoring systemic acetate levels could rescue impaired immune function in obese mice. To test this, obese mice were supplemented with acetate using two independent strategies: chronic oral administration via drinking water (500 mM for 30 days) and i.p. administration (500 mg/kg) prior to and during *A. baumannii* infection (Fig. 2c). Notably, both administration routes elevated circulating acetate to levels approaching those observed in lean mice (Fig. 2d). Furthermore, FFAR2 levels were markedly reduced in infected obese mice (~500 ng/mL) compared with lean controls (~950–1,000 ng/mL). Acetate supplementation significantly restored FFAR2 expression, with oral administration increasing levels to ~1,200 ng/mL and i.p. treatment elevating levels to ~1,600 ng/mL (Fig. 2e). Acetate supplementation partially restored the altered cytokine responses observed in obese mice. IFN-γ levels were markedly reduced in obese mice (~40 pg/mL) compared with lean controls (~80 pg/mL) and increased following acetate supplementation, particularly after i.p. administration (~75 pg/mL), with a more modest increase following acetate administration in drinking water (~65 pg/mL). Similarly, TNF-α levels were reduced in obese mice (~95 pg/mL) relative to lean controls (~150 pg/mL) and increased following acetate supplementation, reaching approximately ~135 pg/mL after i.p. administration and ~105 pg/mL with drinking-water supplementation. IL-6 levels were also reduced in obese mice (~180 pg/mL) compared with lean controls (~300 pg/mL) and were partially restored following acetate treatment (~260 pg/mL for i.p. and ~245 pg/mL for drinking water). Finally, IL-1β levels were reduced in obese mice (~140–150 pg/mL) relative to lean controls (~230–240 pg/mL), with acetate supplementation increasing IL-1β levels to approximately ~200 pg/mL following i.p. administration and ~160 pg/mL following drinking-water supplementation (Fig. 2f through i).

**Fig 3:**
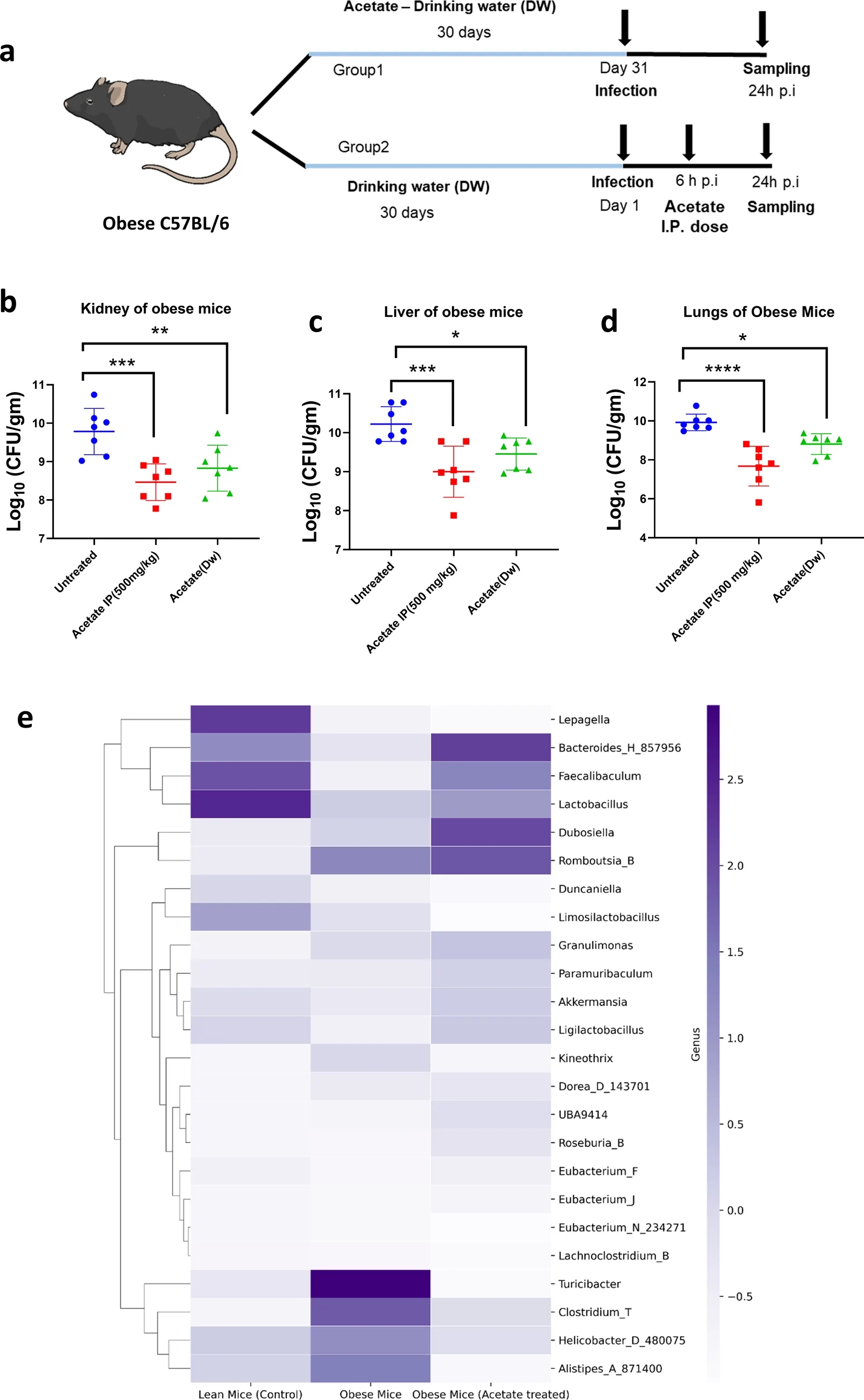
Acetate supplementation reduces bacterial burden and modulates gut microbiota in obese mice. (**a**) Schematic representation of the experimental design. Obese C57BL/6 mice were administered acetate either through DW for 30 days or via a single i.p. injection following infection. Mice were infected at the indicated time points, and organ samples were collected 24 h post-infection for bacterial load determination. (**b–d**) Bacterial burden in (**b**) kidney, (**c**) liver, and (**d**),lungs of obese mice following acetate treatment. Mice were left untreated or treated with acetate (i.p. or DW), infected, and organs were aseptically harvested 24 h post-infection. Tissues were homogenized in sterile phosphate-buffered saline, serially diluted, and plated for c.f.u. enumeration. Bacterial loads are presented as log_10_ c.f.u. per gram of tissue. Each dot represents an individual biological replicate (*n* = 7 mice per group). Data are shown as mean ± s.d. Significance determined by one-way ANOVA followed by Tukey’s multiple comparison test (*P < 0.01; **P < 0.001; ***P < 0.003; ****P < 0.0001; ns, nonsignificant). (**e**) Heat map depicting the relative abundance of gut microbial genera across experimental groups, including lean control mice, obese mice, and obese mice treated with acetate (DW for 30 days). Fecal samples were collected and subjected to 16S rRNA sequencing. Data shown are mean of three independent samples. Taxonomic classification and relative abundance estimation were performed using standard bioinformatics pipelines. Data were normalized and scaled (z-score), and hierarchical clustering was applied to both taxa and samples to visualize compositional differences.

Moreover, acetate supplementation significantly reduced bacterial burden across multiple organs in obese mice, as assessed under the experimental conditions described (Fig. 3a). Intraperitoneal acetate treatment resulted in an approximately 3-log reduction in bacterial load in the kidney, liver, and lung compared with untreated obese controls. Chronic oral supplementation produced a moderate but significant reduction of approximately 1.5–2 log in the kidney, liver, and lung (Fig. 3b through d). Although bacterial clearance was not complete, the substantial reduction in pathogen burden indicates that acetate repletion enhances host antimicrobial competence in obese mice.

### Acetate reprograms the gut microbiome to restore antibiotic efficacy

Although intraperitoneal acetate administration demonstrated strong systemic immunomodulatory effects, chronic oral supplementation models sustained luminal exposure and a host-microbiota metabolic crosstalk. Given that acetate functions both as a microbial metabolite and an ecological substrate within the gut environment, we next examined whether oral acetate supplementation could reshape obesity-associated gut dysbiosis.

16S profiling revealed that HFD-induced obesity was characterized by depletion of SCFA-associated and barrier-protective taxa, alongside enrichment of inflammation-associated genera. Chronic acetate supplementation restored this disruption. At the phylum level, the relative abundance of Bacteroidota, markedly reduced in obese mice, increased following acetate treatment, while the obesity-associated expansion of Bacillota was attenuated (Fig. S4d). In this context, acetate supplementation lowers intestinal pH, which reduces levels of Firmicutes/Bacillota, which are less acid-tolerant as compared to Bacteroidota (41). Additionally, acetate supplementation is also a crucial energy source for increasing the abundance of Bacteroidota, which can compete with Firmicutes for resources (42).

At the genus level, acetate supplementation increased the relative abundance of SCFA-associated and barrier-supportive taxa including *Akkermansia*, *Lactobacillus*, *Limosilactobacillus*, *Ligilactobacillus*, and *Faecalibaculum*. Concurrently, the abundance of inflammation-associated genera such as *Turicibacter*, *Clostridium_T*, *Romboutsia_B*, and *Helicobacter* was reduced (Fig. 3e). These compositional shifts indicate that luminal acetate promotes selective ecological remodeling rather than a global microbiome reversal, consistent with restoration of SCFA-producing taxa.

To functionally validate that acetate-driven microbiome remodeling is sufficient to restore host immune competence and antibiotic responsiveness, we performed fecal microbiota transplantation (FMT) into gut microbiota-depleted recipient mice using donors from lean (control), obese, or acetate-treated obese groups (Fig. S6a).

Compositional analysis of recipient microbiota revealed marked phylum-level alterations reflecting donor status. Recipients of obese microbiota exhibited a significant reduction in *Bacteroidetes* relative abundance compared to lean controls, which was partially restored in recipients of acetate-treated obese microbiota (Fig. S6b). Correspondingly, *Firmicutes* abundance was significantly dysregulated in obese microbiota recipients, and transplantation of acetate-conditioned microbiota normalized this shift relative to obese controls (Fig. S6c). Moreover, the relative abundance of *Akkermansia* was markedly depleted in recipients of obese microbiota but was significantly restored following transfer of acetate-treated microbiota (Fig. S6d), collectively confirming that acetate-associated microbiome remodeling is transmissible through FMT.

We next asked whether FMT leads to compositional shifts in circulating acetate levels. We observed acetate concentrations were significantly reduced in both serum and lung tissues of obese microbiota recipients; however, transplantation from acetate-treated donors restored acetate levels in serum (Fig. S6e) and lung tissue (Fig. S6f). Consistent with recovered SCFA availability, circulating levels of the SCFA receptor FFAR2, which were markedly suppressed in obese microbiota recipients, were fully restored in animals receiving acetate-conditioned microbiota (Fig. S6g), indicating functional recovery of SCFA-mediated immune signaling.

We examined whether these microbiome and metabolite changes were associated with differences in host defense capacity. Following experimental *A. baumannii* infection and tobramycin treatment, recipients of obese microbiota demonstrated significantly impaired bacterial clearance across all examined organ compartments, including the lungs, liver, and kidneys (Fig. S7a through c). Strikingly, transplantation of microbiota derived from acetate-treated obese donors markedly enhanced bacterial clearance in all three tissues across organs, restoring clearance to levels comparable to those observed in lean microbiota recipients. These findings provide causal evidence that acetate supplementation corrects obesity-associated microbial dysbiosis and, critically, that this remodeled microbiome is functionally sufficient to restore host immune competence and tobramycin efficacy during *A. baumannii* infection. The gut microbiome, thus, emerges as a central and transferable intermediary linking host metabolic state, SCFA signaling, and antibiotic performance altogether.

### Acetate enhances tobramycin efficacy against gram-negative pathogens in obesity

**Fig 4:**
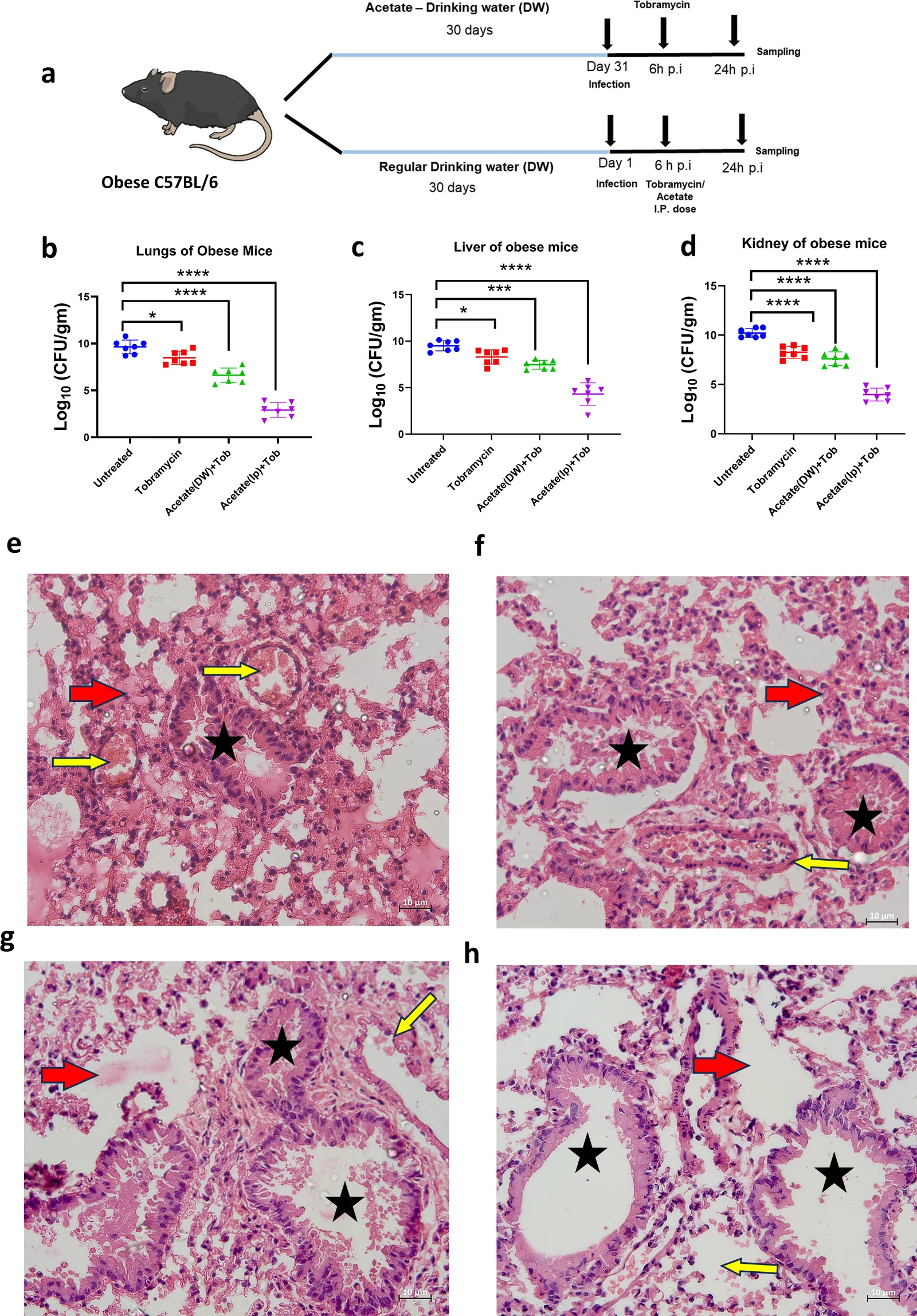
Acetate supplementation enhances antibiotic efficacy in obese mice during systemic infection. (**a**) Schematic representation of the experimental design. Obese C57BL/6 mice were administered acetate via DW for 30 days prior to infection or received acetate in combination with tobramycin via i.p. injection. In the DW (500 mM) group, mice continued acetate supplementation, were infected at day 31, and received tobramycin at 6 h post-infection. In the subsequent group, mice were infected at day 1 with *A. baumannii* 17978 at 10^9^ CFU/mL, followed by antibiotic treatment (tobramycin; 10 mg/kg) along with acetate (500 mg/kg body weight) at 6 h post-infection. Organs were harvested 24 h post-infection for downstream analyses. (**b–d**) Bacterial burden in (**b**) lungs, (**c**) liver, and (**d**) kidneys of obese mice following different treatment regimens. Mice were left untreated or treated with tobramycin alone, acetate plus tobramycin (DW), or acetate plus tobramycin (i.p.). Organs were aseptically harvested 24 h post-infection, homogenized in sterile phosphate-buffered saline, serially diluted, and plated for c.f.u. enumeration. Bacterial loads are presented as log_10_ c.f.u. per gram of tissue. Each dot represents an individual biological replicate (*n* = 7 mice per group). Data are shown as mean ± s.d. Significance determined by one-way ANOVA followed by Tukey’s multiple comparison test (*P < 0.01; **P < 0.001; ***P < 0.003; ****P < 0.0001; ns, nonsignificant). (**e–h**) Representative histopathological analysis of lung tissues from obese mice across the indicated treatment groups (H&E staining, ×400 magnification). Black stars indicate bronchioles, red arrows indicate alveolar spaces, and yellow arrows indicate pulmonary blood vessels. (**e**) Untreated infected lungs show marked inflammatory cellular infiltration surrounding the bronchioles and extending into the adjacent alveolar parenchyma, accompanied by disruption of the normal alveolar architecture. (**f**) Tobramycin-treated lungs show persistent peribronchiolar and parenchymal inflammatory changes, with inflammatory cell accumulation around the bronchioles and within the surrounding lung tissue. (**g**) Acetate (DW) plus tobramycin-treated lungs show partial improvement in pulmonary histology, with comparatively reduced inflammatory involvement around the bronchioles and improved preservation of the surrounding alveolar spaces. (**h**) Acetate (i.p.) plus tobramycin-treated lungs show comparatively better preservation of bronchiolar and alveolar architecture, with reduced inflammatory cellular infiltration in the peribronchiolar and surrounding pulmonary tissue.

We next asked whether restoring acetate availability could overcome obesity-associated tobramycin tolerance. Obese mice were supplemented with acetate either via drinking water (500 mM) or by intraperitoneal administration (500 mg/kg) prior to and during tobramycin treatment following *A. baumannii* infection (Fig. 4a).

Strikingly, acetate supplementation in combination with tobramycin markedly enhanced bacterial clearance across all examined organs compared with tobramycin monotherapy in obese mice. In the lungs, tobramycin alone resulted in approximately ~1 log reduction in bacterial burden, whereas combination therapy produced an approximately 6- to 8-log reduction relative to untreated controls (Fig. 4b). Similar improvements were observed in the liver and kidney, where acetate co-administration significantly amplified tobramycin efficacy, achieving near-complete clearance in this group. Intraperitoneal acetate delivery yielded the most pronounced effect, although chronic oral supplementation also significantly improved antibiotic response (Fig. 4c and d). Histopathological analysis of lethally infected lung tissues revealed that untreated obese mice exhibited severe alveolar consolidation, dense immune cell infiltration, and near-complete loss of alveolar airspace (Fig. 4e). Tobramycin monotherapy showed sustained immune infiltration and tissue damage, which corroborates with the CFU data (Fig. 4f). Both acetate delivery routes attenuated pulmonary pathology, whereas drinking water acetate reduced peribronchiolar infiltration, while intraperitoneal acetate more effectively preserved alveolar patency with broader inflammatory resolution in combination with tobramycin (Fig. 4g and h). Notably, combined intraperitoneal acetate and tobramycin produced the most favorable histological outcome, with maximal restoration of alveolar architecture and minimal interstitial pathology, indicating that acetate synergistically resolves infection-driven pulmonary inflammation beyond the bactericidal action of tobramycin alone (Fig. 4h).

To determine whether this potentiating effect extended beyond *A. baumannii*, we evaluated the combination therapy in obese mice infected with *P. aeruginosa*. Consistent with the above findings, acetate co-administration significantly enhanced tobramycin-mediated bacterial clearance in lung, liver, and kidney tissues, achieving an approximately 7-log reduction compared with untreated controls (Fig. S8a through c).

Collectively, these data demonstrate that restoring systemic acetate availability reverses obesity-associated aminoglycoside tolerance and improves tobramycin efficacy against these pathogens.

### Acetate potentiates tobramycin by rewiring bacterial membrane energetics

Our *in vivo* data showed that acetate alone improved host immune responses, whereas tobramycin monotherapy was less effective in obese mice, consistent with host-dependent phenotypic tolerance. Notably, co-administration of acetate and tobramycin restored antibiotic efficacy, suggesting that acetate may enhance aminoglycoside activity. Based on this observation, we hypothesized that acetate alters bacterial physiological states in a manner that enhances susceptibility to tobramycin, potentially by facilitating antibiotic uptake or restoring antibiotic-sensitive metabolic activity.

**Fig 5:**
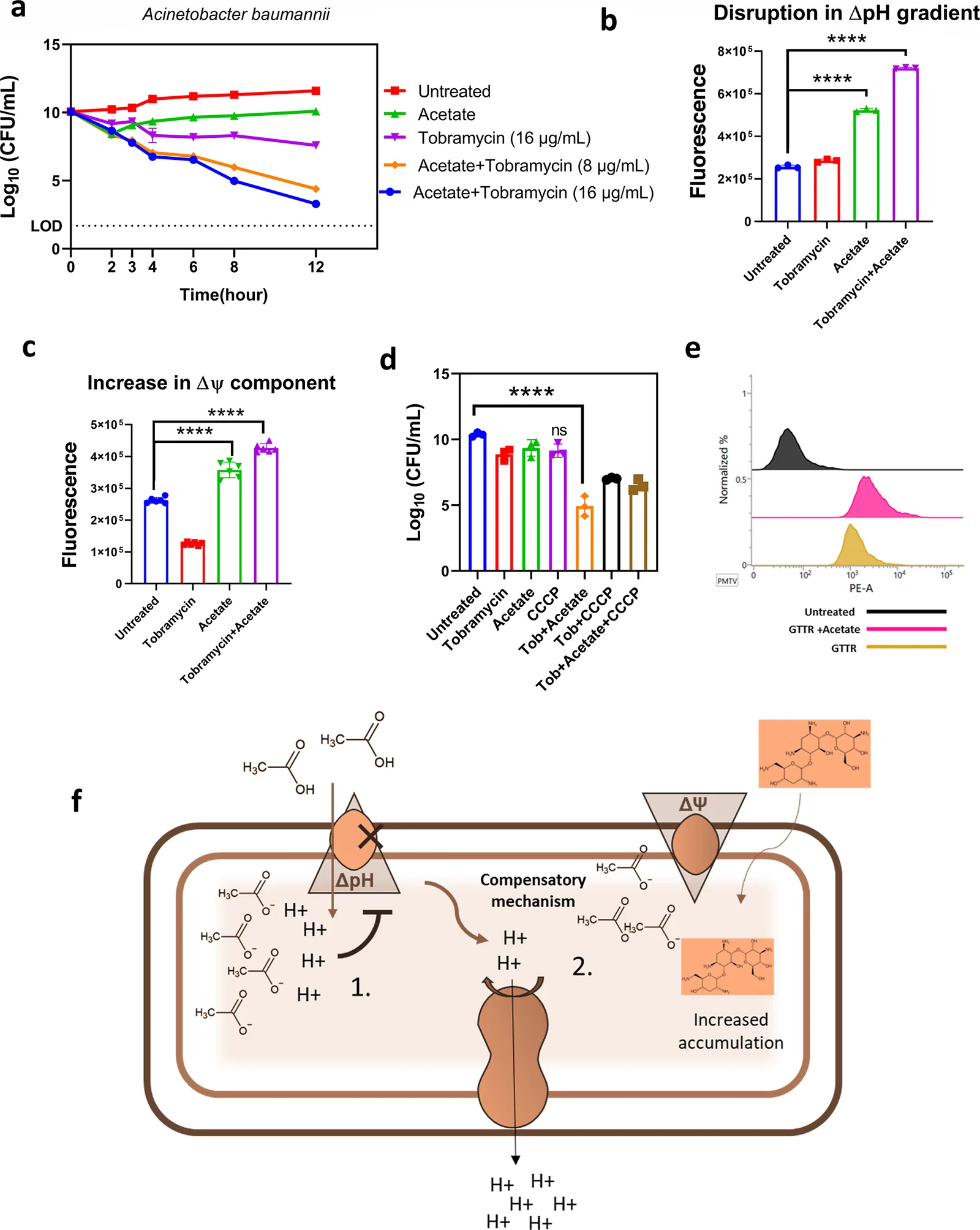
Acetate enhances tobramycin efficacy by altering bacterial membrane energetics. (**a**) Bacterial cultures were treated with acetate (20 mM), tobramycin (16 μg/mL), or a combination of acetate and tobramycin, and viable counts were determined at the indicated time points by plating serial dilutions on agar for c.f.u. enumeration. Data are presented as log_10_ c.f.u./mL. The dashed line indicates the limit of detection (LOD). (**b**) Disruption of the transmembrane pH gradient (ΔpH) following treatment. Changes in ΔpH were assessed using a pH-sensitive fluorescent probe (ACMA), and fluorescence intensity was quantified using a microplate reader. Data are presented as normalized fluorescence. (**c**) Increase in membrane potential (Δψ) component of the proton motive force (PMF) upon treatment. Membrane potential was measured using a Δψ-sensitive fluorescent dye (DiSC3), and fluorescence intensity was recorded and normalized across conditions. (**d**) Bacterial cultures were grown to exponential phase and subjected to the indicated treatments: untreated control, tobramycin (8 μg/mL), acetate (20 mM), CCCP (128 μg/mL), and their combinations (Tob+Acetate, Tob+CCCP, and Tob+Acetate+CCCP). Following treatment, cultures were incubated at 37°C with shaking for 4 h. Viable bacterial counts were determined by plating and expressed as log_10_ CFU/mL. Data represent [mean ± SD/ SEM, from *n* = 3 independent experiments]. (**e**) Flow cytometry analysis of intracellular accumulation of tobramycin. Bacterial cells were treated under indicated conditions, stained using a fluorescent reporter or labeled antibiotic analog, and analyzed by flow cytometry. Representative histograms show fluorescence intensity (PE-A), indicating intracellular drug accumulation. GTTR, gentamicin-Texas Red conjugate. (**f**) Proposed mechanistic model illustrating acetate-mediated potentiation of aminoglycoside activity. Acetate disrupts the ΔpH component of the proton motive force, triggering compensatory increases in Δψ. This shift enhances aminoglycoside uptake across the bacterial membrane, leading to increased intracellular accumulation and improved bactericidal activity. Data in panels **b and c** are shown as mean ± s.d. from independent experiments (*n* = 3). Significance determined by one-way ANOVA followed by Tukey’s multiple comparison test (*P < 0.01; **P < 0.001; ***P < 0.003; ****P < 0.0001; ns, nonsignificant).

To test this, we first examined killing kinetics in *A. baumannii,* where tobramycin monotherapy resulted in an approximate 3–4 log_10_ reduction in CFU over 12 h relative to untreated controls (Fig. 5a). In contrast, co-treatment with acetate (5 mM) produced a 7–8 log fold decrease relative to untreated bacteria (Fig. 5a). Acetate monotherapy produced minimal changes in bacterial burden (<1 log fold difference), indicating that its effect is contingent upon antibiotic exposure. We next extended this analysis to other clinically relevant gram-negative pathogens, including *E. coli* 25922, *P. aeruginosa*, and *K. pneumoniae* (Fig. S8d and e), which is consistent with previous findings (11). In each case, the acetate-tobramycin combination produced substantially greater reductions in CFU compared with antibiotics alone, demonstrating that acetate-mediated potentiation is not restricted to a single species.

Since aminoglycoside uptake is driven primarily by the bacterial transmembrane electrical potential (Δψ) (43), we hypothesized that acetate, by virtue of its membrane-permeant weak acid properties, could redistribute PMF components in a manner that amplifies antibiotic entry. To test this, we independently measured both PMF components following combination treatment. This significantly disrupted the transmembrane ΔpH gradient while inducing a compensatory elevation in Δψ (Fig. 5b and c), which is consistent with previous findings (44, 45). To establish whether this potentiation requires intact membrane energetics, we pharmacologically dissipated PMF using the protonophore CCCP and assessed the survival. Disruption of PMF abolished the enhanced bactericidal activity observed under acetate co-treatment, restoring bacterial survival to levels comparable to antibiotic treatment alone. These findings demonstrate that acetate-mediated potentiation of aminoglycoside activity is critically dependent on PMF (Fig. 5d).

Flow cytometry analysis using fluorescently labeled gentamicin-Texas Red further confirmed markedly enhanced intracellular antibiotic accumulation in combination-treated bacteria (Fig. 5e). Here, gentamicin served as a fluorescently traceable aminoglycoside surrogate to directly monitor antibiotic uptake. These findings can be mechanistically explained by the weak acid properties of acetate which upon freely diffusing across the inner membrane as acetic acid dissociates intracellularly, releasing protons that collapse the ΔpH gradient. To restore PMF homeostasis, bacteria initiates a compensatory hyperpolarization of Δψ, which is precisely the electrochemical component that drives electrophoretic aminoglycoside entry. This inadvertently amplifies intracellular aminoglycoside accumulation and potentiates its bactericidal activity (Fig. 5f).

In addition to enhancing antibiotic uptake, acetate-tobramycin co-treatment profoundly altered bacterial metabolic and redox homeostasis. Resazurin-based metabolic activity assays revealed a significant reduction in cellular reductive/metabolic activity in acetate-treated and combination groups compared with controls (Fig. S9a), indicating disruption of redox balance. Consistently, ATP levels were markedly decreased, with the most pronounced depletion observed under combination treatment (Fig. S9b), reflecting severe metabolic stress. This metabolic perturbation was accompanied by a substantial increase in intracellular reactive oxygen species (ROS), as evidenced by elevated fluorescence intensity (Fig. S9c) measured using the ROS-sensitive dye DCFH-DA, along with a pronounced rightward shift in flow cytometry profiles (Fig. S9d), particularly in the acetate-tobramycin-treated group. Functional validation using oxidative stress-deficient mutants further supported this mechanism as *ΔsodB*::Tn and *ΔsodC* strains exhibited significantly enhanced susceptibility to combination treatment compared with wild-type bacteria (Fig. S9e) (46). The phenotype was reversed upon using complemented strains of both the deletion mutants. In addition, treatment with vitamin C, at a concentration that did not independently inhibit bacterial growth, partially attenuated the bactericidal activity of the acetate-tobramycin combination (Fig. S9f). Together, these findings are consistent with the contribution of oxidative stress to the enhanced antibacterial activity of the acetate-tobramycin combination.

Mechanistically, these findings suggest that acetate drives a metabolic imbalance that disrupts electron transport and redox cycling, leading to leakage of electrons and excessive ROS generation. In the presence of tobramycin, which already perturbs membrane potential and translational fidelity, this heightened oxidative stress overwhelms bacterial antioxidant defense, resulting in oxidative damage to intracellular compartments, collapse of cellular energetics, and ultimately bacterial cell death.

## DISCUSSION

The central implication of this work is that host metabolic state functions as an independent determinant of antibiotic efficacy during infection caused by bacterial pathogens. This is consistent with the broader concept of “physiological tolerance,” wherein the metabolic and immune state of the host, rather than the bacterium alone, governs the outcome of antibiotic treatment (10). Moreover, as per our findings, the inflammatory pre-conditioning in obese hosts does not confer enhanced resistance to acute infection. Instead, it impairs the capacity for regulated immune surge that is essential for effective pathogen clearance. A key mechanistic basis is that obesity impairs neutrophil chemotaxis and survival, limiting effective recruitment to sites of infection and weakening early bacterial clearance (47). At the molecular level, chronic metabolic inflammation in obesity induces persistent activation of NF-κB and STAT3 signaling pathways (48), resulting in elevated basal cytokine production and immune desensitization. Obesity-associated hyperglycemia and excess free fatty acids further disrupt TLR-mediated signaling (49), impair inflammasome activation, and alter macrophage and neutrophil metabolic programming, which may compromise coordinated early cytokine responses and bacterial sensing. In addition, altered adipokine signaling, particularly increased leptin and reduced adiponectin levels, promotes prolonged inflammatory activation while impairing efficient resolution of inflammation (50). Such defect in frontline innate defense creates a permissive infection microenvironment in which bacteria persist despite antibiotic exposure, thereby promoting antibiotic tolerance independent of the bacterial genotype (10). The impaired induction of TLR4, downstream cytokines, and neutrophil-recruiting chemokines observed in obese mice is consistent with a model in which chronic inflammation exhausts or desensitizes the immune response, leaving the host unprepared for acute bacterial infection (51, 52). Consistent with this, recent evidence demonstrates that gut microbiome disruption suppresses neutrophil-mediated nutritional immunity including lipocalin-2 and calprotectin expression during *A. baumannii* infection, causally linking dysbiosis to impaired innate defense at the infection site (53). While this work established microbiome-immune crosstalk as a driver of bacterial dissemination and burden, our findings extend this axis to antibiotic pharmacodynamics, demonstrating that obesity-driven dysbiosis and acetate depletion generate a tolerogenic infection microenvironment that antibiotic treatment alone cannot overcome. Our findings support the concept that the host metabolic and immune environment can independently influence antibiotic treatment outcomes; however, dedicated experiments examining bacterial killing kinetics, survival dynamics, and regrowth of antibiotic-exposed bacteria recovered from infected hosts will be required to distinguish tolerance from persistence in this setting.

Our findings demonstrate that acetate supplementation restores immune competence, enhancing neutrophil-associated responses and significantly improving bacterial clearance and antibiotic efficacy in obese hosts. Acetate is the simplest SCFA, consisting of a two-carbon structure (CH_3_COO^−^), and is a major end product of anaerobic microbial fermentation in the gut (54). It is primarily generated by commensal bacteria, particularly members of the Bacteroidetes phylum, through the breakdown of dietary fibers and resistant starches, making it one of the most abundant circulating SCFAs in the host (34). Consistent with this, our findings showing reduced *Bacteroidetes* abundance in obese hosts provide a plausible explanation for the observed decline in systemic acetate levels. Beyond its endogenous production, acetate has established clinical applications where it is routinely used as a buffering agent in intravenous fluids (e.g., sodium acetate in parenteral nutrition) and in dialysis solutions (55), where it contributes to systemic acid-base balance. Prior work has demonstrated that microbiota-derived acetate is a key regulator of immune cell metabolic fitness and function (56). It has been shown in germ-free conditions that reduced acetate availability leads to profound defects in immune cell metabolism, including impaired mitochondrial function and altered gene expression, resulting in an immature and functionally compromised immune phenotype (56). Importantly, exogenous acetate supplementation was sufficient to restore metabolic activity and normalize immune signaling pathways such as NF-κB, highlighting acetate as a critical link between the gut microbiome and host immune competences (57, 58).

**Fig 6:**
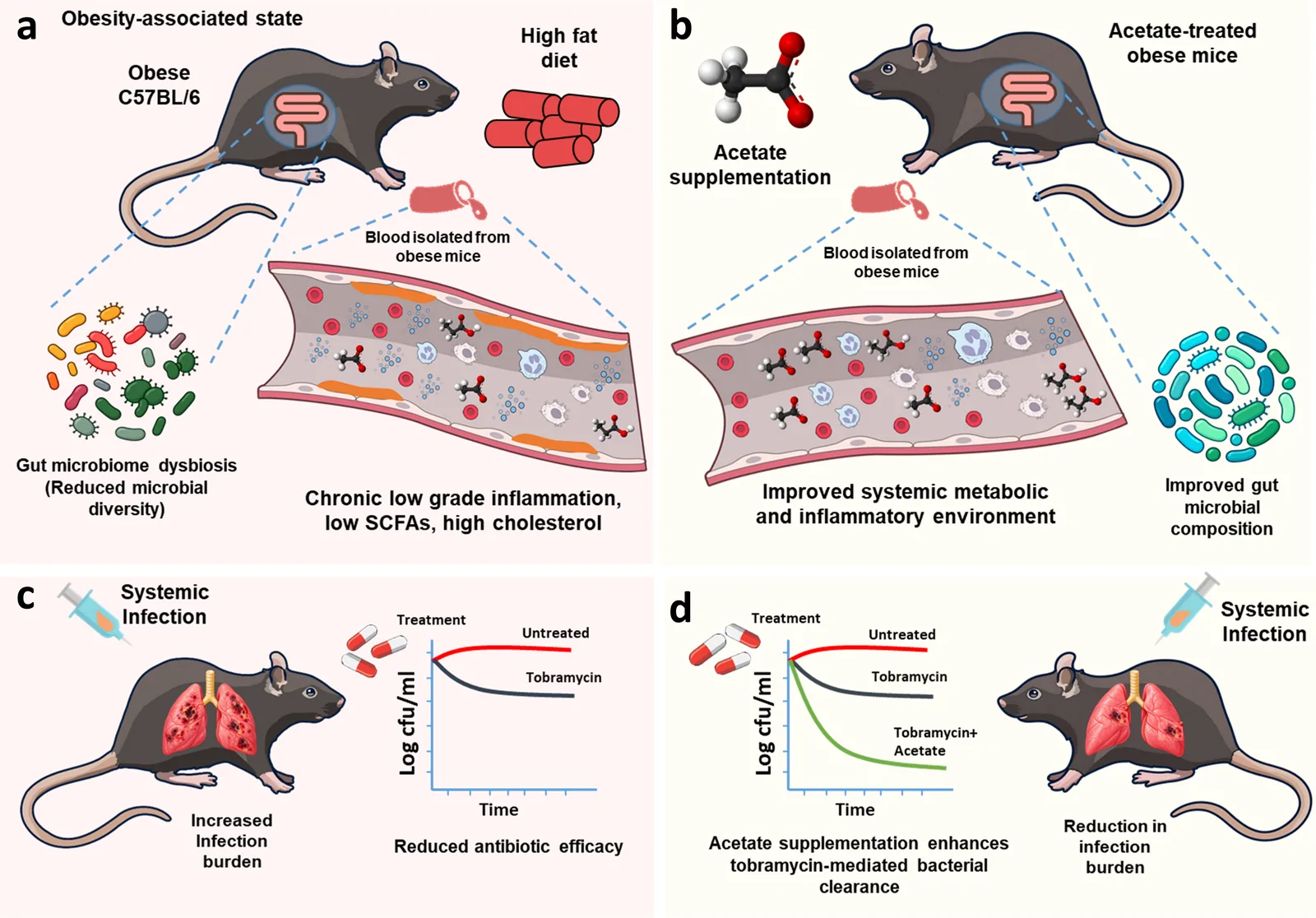
Proposed model illustrating the effects of acetate supplementation on obesity-associated infection and antibiotic efficacy. (**a**) High-fat diet-induced obesity is associated with gut microbiome dysbiosis, characterized by reduced microbial diversity, along with reduced SCFA availability, chronic low-grade inflammation, and an altered systemic metabolic environment. (**b**) Acetate supplementation is associated with improvements in gut microbial composition and the systemic metabolic and inflammatory environment in obese mice. (**c**) During systemic infection, the obesity-associated host environment is associated with increased bacterial burden in lungs and reduced efficacy of tobramycin, resulting in less efficient bacterial clearance. (**d**) Acetate supplementation enhances tobramycin-mediated bacterial clearance, leading to improved treatment response and a reduction in bacterial burden during obesity-associated infection.

Aminoglycosides, such as tobramycin, are widely used as first-line or frontline agents against severe gram-negative infections, including *A. baumannii* and *P. aeruginosa*, owing to their rapid bactericidal activity (59, 60). However, their efficacy is highly dependent on bacterial metabolic state and environmental conditions, particularly extracellular pH and membrane potential, which govern antibiotic uptake (40). Under conditions of host immune dysfunction such as those observed in obesity, reduced inflammatory activation and impaired tissue microenvironments can limit aminoglycoside uptake, thereby promoting phenotypic tolerance without the acquisition of genetic resistance (10). In this context, the central clinically relevant implication of our work is that acetate supplementation modulates the host response by restoring immune and metabolic signaling, including FFAR2-associated signaling and inflammatory homeostasis, thereby improving the environment for bacterial clearance. In parallel, acetate can act directly on *A. baumannii* to alter bacterial metabolism and membrane energetics, including disruption of the ΔpH component of the proton motive force and a compensatory increase in membrane potential, which enhances intracellular aminoglycoside accumulation and thereby potentiates antibiotic activity. Notably, the combination of acetate with tobramycin significantly improves bacterial clearance, indicating that modulation of the host-microbiome-metabolite axis can overcome phenotypic tolerance in obese hosts (Fig. 6. This approach highlights a promising therapeutic strategy to mitigate antibiotic failure by reinforcing host-driven mechanisms rather than directly targeting bacterial resistance pathways.

## MATERIALS AND METHODS

### Bacterial strains and reagents

The strains *A. baumannii* ATCC 17978, *K. pneumoniae* ATCC 700698, and *P. aeruginosa* PA01 and *E. coli* 25922 were used for the study. The complete lists of strains and plasmids used in the study are provided in Tables S1 and S3, respectively. The growth medium was cation-adjusted Mueller-Hinton broth (CAMHB), Luria Bertani (LB) broth, LB agar, or Leeds *Acinetobacter* agar. Antibiotics used in this study were procured from Sigma-Aldrich and Tokyo Chemicals Industries Limited (TCI) and stored as prescribed. Fluorescent dyes and indicators, including DCFH-DA, DiSC3(5), DiBAC4(3), and resazurin, were also purchased from Sigma-Aldrich and handled as per the manufacturer’s instructions.

### Minimum inhibitory concentration (MIC) determination

MICs were determined using the standard broth microdilution method in cation-adjusted Mueller-Hinton broth, following CLSI guidelines. Briefly, mid-log phase bacterial cultures were diluted to ~5 × 10⁵ CFU/mL and exposed to serial twofold dilutions of antibiotics. Plates were incubated at 37°C for 16–18 h, and MIC was defined as the lowest concentration with no visible bacterial growth. The MIC values are provided in Table S2.

### Animal studies

Female C57BL/6 mice (6–8 weeks old) were maintained under specific pathogen-free conditions in individually ventilated cages (IVC) with controlled temperature, humidity, and a 12-h light/dark cycle. To induce obesity, mice were fed a HFD (60% kcal from fat, Research diet) for 70 days, while control mice received standard chow. Body weight and metabolic parameters were monitored throughout the study.

Detailed methodologies for blood collection, establishment of the infection model, *in vivo* estimation of biochemical parameters (61), and qPCR analysis of organ homogenates are provided in the Supplementary Information.

### Antibiotic tolerance

Antibiotic dosing was adjusted based on body weight to ensure comparable pharmacological exposure between lean and obese mice as described earlier (62). The detailed methodology is provided in the Supplemental Information.

### Tobramycin quantification

Tobramycin concentrations in biological samples were quantified using a competitive ELISA kit (Cat. No. DEIA-BY018) from Bioassay Technology Laboratory, according to the manufacturer’s instructions. Briefly, serum samples (collected 30 min post 10 mg/kg i.p. dose) and standards were added to precoated ELISA plates and incubated with biotinylated antibody, followed by streptavidin-horseradish peroxidase conjugate. After washing, the TMB substrate was added for color development, and the reaction was terminated using stop solution. The absorbance was measured at 450 nm using a microplate reader, and tobramycin concentrations were calculated from a standard curve generated using kit-provided standards. Due to the competitive ELISA format, the signal intensity was inversely proportional to tobramycin concentration. The assay sensitivity was 38.05 pg/mL, with intra- and inter-assay coefficients of variation <10%.

### Fecal sample collection and 16S profiling

Fresh fecal samples were collected under sterile conditions and immediately stored at −80°C until processing. Microbial DNA was extracted using the QIAamp PowerFecal Pro DNA Kit according to the manufacturer’s instructions. Detailed methodologies for metagenomic sequencing and downstream analyses are provided in the Supplemental Information.

### Fecal microbiome transplantation

The transplantation experiment was done as described previously (10). Detailed procedures for donor fecal sample preparation, recipient pre-treatment, transplantation protocol, and post-transplantation analyses are provided in the Supplemental Information.

### Acetate supplementation and antibiotic treatment in murine systemic infection

Diet-induced obese (DIO) C57BL/6 mice were used for infection studies with *Acinetobacter baumannii* ATCC 17978 and *Pseudomonas aeruginosa* (PAO1). Detailed procedures for acetate supplementation, bacterial challenge, antibiotic treatment, sample collection, and assessment of treatment efficacy are provided in the Supplemental Information.

### Assay for *in vitro* antibiotic tolerance

*In vitro* tolerance assay was done as described previously (11). The detailed experimental procedure is provided in the Supplemental Information.

### Membrane potential assays

Membrane potential was assessed using the fluorescent dyes DiSC₃ (5) and ACMA. The detailed methodology for the assay is provided in the Supplemental Information.

### Gentamicin-Texas Red uptake and flow cytometry

The assay was done as described previously (63). The detailed methodology is provided in the methods section of the Supplemental Information.

### Metabolic assays

The assay was done as previously described (64). The detailed methodology is provided in the methods section of the Supplemental Information.

### Reactive oxygen species measurement

The assay was done as previously described (64). The detailed methodology is provided in the methods section of the Supplemental Information.

### Generation of clean knockout strain of the *sodC* gene

The assay was done as described previously (63). The detailed methodology is provided in the methods section of the Supplemental Information.

### Statistical analysis

Statistical analysis was performed using GraphPad Prism v.8.3.0 for Windows. All data are presented as mean ± s.d. unless otherwise indicated. Unless noted, unpaired two-tailed Student’s *t*-tests were used to evaluate differences between two groups, and nonparametric one-way ANOVA was performed to determine differences among multiple groups.

## Data Availability

The sequencing data generated in this study have been deposited in the NCBI Sequence Read Archive (SRA) under BioProject accession number PRJNA1528945. All raw sequencing data are publicly available through the NCBI SRA. All other data are available upon request from the corresponding author.

## SUPPLEMENTAL MATERIAL

Supplemental Information mbio.01630-26-s0001.docx Supplemental methods, figures, and tables.

ASM does not own the copyrights to Supplemental Material that may be linked to, or accessed through, an article. The authors have granted ASM a non-exclusive, world-wide license to publish the Supplemental Material files. Please contact the corresponding author directly for reuse.
