# Supplementary material for: Acetate modulates host physiology and bacterial energetics to improve antibiotic efficacy during obesity-associated infection
Source: mBio. 2026 Oct 16;17(11):e01630-26. doi: 10.1128/mbio.01630-26 (PMC13753818; doi:10.1128/mbio.01630-26)
Supplement: Supplementary file 1 [file mbio.01630-26-s0001.docx]

**Acetate modulates host physiology and bacterial energetics to improve antibiotic efficacy during obesity-associated infection**

Arsalan Hussain^1^, Prateek Bhardwaj^1^, Ashish Kumar Ray^1^, Aakash Singh^1^ , Abhiroop Sett^1,a^, Ranjana Pathania^1,#^

^1^Department of Biosciences and Bioengineering, Indian Institute of Technology Roorkee, Roorkee 247667, India

Running head: Acetate alleviates antibiotic tolerance in obesity

^#^ Address correspondence to Ranjana Pathania, [ranjana.pathania@bt.iitr.ac.in](mailto:ranjana.pathania@bt.iitr.ac.in)

^a^Present address: Department of Microbiology and Molecular Genetics, Institute for Medical Research Israel-Canada, Faculty of Medicine, The Hebrew University of Jerusalem, Jerusalem, Israel

**Supplementary methods**

**Blood isolation and infection model**

For establishment of systemic infection, mice were challenged intraperitoneally (i.p.) with *A. baumannii* ATCC 17978 VU at an inoculum of 1 × 10⁷ CFU suspended in 100 µL of sterile phosphate-buffered saline (PBS). Twenty-four hours post-infection, mice were anesthetized using ketamine (75 mg/kg body weight, i.p.) and xylazine (16 mg/kg body weight, i.p.) and blood samples were collected by retro-orbital puncture. The mice were subsequently euthanized, and the lungs, liver, and kidneys were aseptically harvested. Excised organs were processed immediately for downstream analyses, including determination of bacterial burden and immunological analyses, as described in the respective sections. All organ bacterial burden measurements presented for the acute systemic infection model were performed 24 h post-infection.

**Estimation of cholesterol, triglyceride and cytokine levels by ELISA.**

Serum levels of cytokines, metabolic markers, and host receptors were quantified using commercially available ELISA kits. Kits for IL-1β (Cat. No. EM0109), IL-6 (EM0121), IL-10 (EM0100), IFN-γ (EM0093), TNF-α (EM0183), total cholesterol (EM2131), triglycerides (EM2179) and PTX3 (EM0161) were purchased from Wuhan Fine Biotech Co., Ltd. The Mouse *FFAR2* (Free Fatty Acid Receptor 2) ELISA kit was obtained from ELK Biotechnology. All assays were performed according to the manufacturers’ instructions. Briefly, serum samples were diluted (1:1000) and added to ELISA plate wells, followed by incubation at 37 °C for 90 min to allow antigen binding. Plates were washed with 1× PBST to remove unbound components. Subsequently, biotinylated detection antibody was added and incubated at 37 °C for 60 min. After washing, 100 μL of streptavidin–biotin–peroxidase complex (SABC) was added and incubated for 30 min at 37 °C. Plates were washed again, and 90 μL of TMB substrate was added, followed by incubation in the dark at 37 °C for 15 min for color development. The reaction was terminated using 50 μL of stop solution, resulting in a yellow-colored product. Absorbance was measured at 450 nm using a Synergy H1 microplate reader. Concentrations of analytes were calculated based on standard curves generated using known standards provided in the kits.

**Quantitative PCR (qPCR) from organ homogenates**

Organs were aseptically collected, immersed immediately in RNAlater (QIAGEN, Germantown, MD), and stored at −20°C until extraction. The organs were initially crushed, followed by homogenization using silica beads in a NEUATION iRuptor 24P tissue homogenizer. Following homogenization, TRIzol reagent (Thermo Fisher Scientific) was added to the tissue homogenates for total RNA extraction according to the manufacturer's instructions followed by DNAse treatment. cDNA synthesis was performed using the PrimeScript 1st Strand cDNA Synthesis Kit (Takara) according to the manufacturer's instructions. Quantitative PCR was performed using SYBR Green Master Mix on a QuantStudio 5 Real-Time PCR System (Thermo Fisher Scientific) using a three-step amplification program. Gene expression levels were normalized to the housekeeping gene *Gapdh* and calculated using the 2^−ΔΔCt method. The primer sequences used for qPCR are provided in Supplementary Table S4.

**Antibiotic tolerance**

Lean and diet-induced obese mice were treated with polymyxin B, meropenem, rifampicin, or tobramycin, with all antibiotics administered intraperitoneally (i.p.). Lean mice had an average body weight of 25 g, which was used as the reference ideal body weight (IBW), whereas obese mice had an average total body weight (TBW) of approximately 35 g. For polymyxin B and tobramycin, adjusted body weight (AdjBW) was used for dosing in obese mice to account for differences in body composition (1)(2)(3)(4). AdjBW was calculated using the established equation AdjBW = IBW + 0.4 × (TBW − IBW). Thus, for obese mice, AdjBW was calculated as 25 + [0.4 × (35 − 25)] = 29 g, whereas a body weight of 25 g was used for lean mice. Polymyxin B was administered at 5 mg/kg and tobramycin at 10 mg/kg using the respective dosing weights. Meropenem was administered at 100 mg/kg and rifampicin at 25 mg/kg based on total body weight. All antibiotics were freshly prepared in sterile PBS or saline and administered intraperitoneally at the indicated time points following infection. At the indicated endpoint following infection and antibiotic treatment, lungs, liver, and kidneys were aseptically collected from individual mice and homogenized in sterile PBS. Serial dilutions of tissue homogenates were plated on appropriate agar plates, and bacterial colonies were enumerated following incubation. The bacterial burden for each individual animal was expressed as CFU per organ (or CFU/g tissue, if tissue weights were used).

To compare antibiotic efficacy between lean and diet-induced obese mice while accounting for differences in baseline bacterial burden, antibiotic-mediated killing was expressed as log₁₀ reduction in CFU relative to the corresponding untreated control group within each dietary condition. The calculation was performed as follows:

Log_10_ CFU reduction = Log_10_ (mean CFU of corresponding untreated group) - Log_10_ (CFU of individual treated mouse)

**Acetate quantification and administration**

Acetate levels in serum and tissue samples were quantified using a colorimetric acetate assay kit (Sigma-Aldrich) following manufacturer instructions. For *in vivo* supplementation, potassium acetate was administered either via intraperitoneal injection or through drinking water. For i.p. administration, mice received acetate at a dose of 500 mg/kg body weight. For oral delivery, acetate was supplemented in drinking water (500 mM) ad libitum for 30 days.

**Fecal sample collection and 16S profiling**

DNA quantity and quality were assessed using the NanoDrop One and Qubit Fluorometer. The V3–V4 region of the bacterial 16S rRNA gene was amplified following the Illumina Inc.16Somic library preparation was done using sequences - Fresh fecal samples were collected under sterile conditions and immediately stored at −80 °C until processing. Microbial DNA was extracted using the QIAamp PowerFecal Pro DNA Kit according to the manufacturer’s instructions. DNA quantity and quality were assessed using the NanoDrop One and Qubit Fluorometer. The V3–V4 region of the bacterial 16S rRNA gene was amplified following the Illumina Inc.16S metagenomic library preparation protocol (FP:5′TCGTCGGCAGCGTCAGATGTGTATAAGAGACAGGTGYCAGCMGCCGCGGTAA-3′; and RP:5′GTCTCGTGGGCTCGGAGATGTGTATAAGAGACAGGGACTACNVGGGTATCTAAT-3′). Amplicon libraries were generated using 12.5 ng input DNA, followed by bead purification and a second PCR to incorporate dual indices and sequencing adapters. Final libraries were purified, quantified using Qubit, and fragment size distribution was assessed using a DNA 3K marker system. Libraries were pooled in equimolar concentrations and subjected to high-throughput paired-end sequencing. Raw sequencing data were imported into QIIME2 using a manifest file, and read quality was assessed. Sequence denoising, merging of paired-end reads, chimera removal, and primer trimming were performed using the DADA2 plugin. Reads were trimmed (17 bp forward, 21 bp reverse) to remove primer sequences and truncated at 280 bp (forward) and 270 bp (reverse) to eliminate low-quality regions. Quality filtering was based on expected error (maxEE) and Phred score criteria. Denoising resulted in the identification of 2,712 amplicon sequence variants (ASVs) with a mean length of 405.01 bp. Taxonomic assignment was performed using the Greengenes2 backbone reference database (2024.09). Closed-reference clustering was conducted using the “non-v4-16s” plugin with q2-vsearch, resulting in 756 ASVs with a mean length of 411.29 bp.

**Fecal microbiome transplantation**

Briefly, recipient mice were depleted of gut microbiota by administration of an antibiotic cocktail in drinking water (streptomycin, 1 mg mL⁻¹; ampicillin, 0.5 mg mL⁻¹; gentamicin, 1 mg mL⁻¹; and vancomycin, 0.5 mg mL⁻¹). Antibiotic-containing water was refreshed every 2 days and discontinued 2 days prior to fecal microbiota transplantation. To confirm depletion of the recipient gut microbiota, fecal samples were collected immediately before and after the 7-day antibiotic cocktail treatment. Culturable bacterial burden was assessed by direct plating of serially diluted fecal samples, and fecal DNA was analyzed by quantitative PCR (qPCR) to determine the relative abundance of *Bacteroidetes* and *Firmicutes*. Taxon-specific 16S rRNA gene levels were normalized to universal bacterial 16S rRNA gene levels and expressed using the 2⁻ΔCt method, where ΔCt = Ct(taxon-specific target) − Ct(universal 16S rRNA gene). Mice were then randomized into experimental groups (Group 1: lean microbiota recipients; Group 2: obese microbiota recipients; Group 3: obese microbiota recipients + acetate supplementation). Fresh fecal samples (~100 mg) from donor mice were resuspended in 1.5 mL sterile anaerobic PBS, homogenized, and centrifuged at 3,000 × g. The resulting supernatant was administered to recipient mice via oral gavage (200 μL per mouse) daily for 14 days. Following transplantation, mice were maintained on a standard diet and regular drinking water for 43 days prior to downstream analyses and infection experiments. Microbial reconstitution was assessed by quantitative PCR (qPCR) targeting major bacterial taxa (*Bacteroidetes*, *Firmicutes*, and *Akkermansia*) using taxon-specific primers (listed in Supplementary Information) and expressed as relative abundance using the 2⁻ΔCt method described above. Functional outcomes were evaluated by measuring circulating acetate levels, expression of free fatty acid receptor 2 (*FFAR2*), and efficacy of tobramycin post infection with *A. baumannii*.

**Acetate supplementation and antibiotic treatment in murine systemic infection**

Two acetate dosing regimens were evaluated. In the first group, potassium acetate was administered in drinking water (500 mM) for 30 days prior to infection, while control mice received regular drinking water. On day 31, mice were infected with ~1 × 10⁷ CFU of either strain. Tobramycin (10 mg kg⁻¹, i.p.) was administered at 6 h post-infection, and organs were harvested at 24 h post-infection for CFU enumeration. In a second experimental setup, DIO mice were infected with a similar inoculum and, at 6 h post-infection, treated with acetate (i.p.), tobramycin (i.p.), or their combination. Organs were collected at 24 h post-infection, and bacterial burden was determined by CFU enumeration.

**Assay for in-vitro tolerance.**

Briefly, stationary-phase cultures of *A. baumannii* ATCC 17978 VU, *K. pneumoniae*, *P. aeruginosa*, and *E. coli* were prepared by overnight growth in LB medium. Cells were harvested, washed with PBS, and resuspended in carbon-free M9 minimal medium.

Bacterial cultures were exposed to tobramycin at 16 µg/ml and incubated for 12h. Where indicated, potassium acetate (20 mM) was added to assess its effect on antibiotic tolerance. At specified time points, cells were washed with PBS, serially diluted (1:10) in PBS, and plated on LB agar for CFU enumeration following incubation. The *A. baumannii sodB::Tn and sodC* mutant and complemented strain were processed under identical conditions where indicated. acterial cultures were exposed to tobramycin (16 µg mL⁻¹), with or without potassium acetate (20 mM), and incubated for 8 h. Cells were subsequently washed with PBS, serially diluted (1:10) in PBS, and plated on LB agar for CFU enumeration. Bacterial survival was calculated as percentage survival relative to the viable bacterial population immediately before treatment (0 h) as CFU₈ₕ/CFU₀ and expressed as log₁₀(%survival).

**Membrane potential assays**

Primary cultures were grown overnight in LB medium, diluted 1:100 into fresh medium, and grown to mid–late exponential phase (OD₆₀₀ ≈ 2.0). Cells were harvested and washed three times with PBS. Bacterial suspensions were divided into treatment groups: untreated control, tobramycin (16 μg mL⁻¹), acetate (20 mM), and a combination of acetate and tobramycin. Cultures were incubated at 37 °C for 4 h, followed by washing and resuspension in PBS. For the DiSC₃(5) assay, cells were incubated with 2 μM dye for 30 min, and endpoint fluorescence was measured at 620 nm using black 96-well plates. For the ACMA assay, cells were incubated with 5 nM ACMA for 15 min, and endpoint fluorescence was measured at excitation/emission wavelengths of 419/483 nm. Changes in fluorescence intensity were used as an indicator of alterations in membrane potential and proton motive force.

**Gentamicin–Texas Red (GTTR) uptake and flow cytometry**

Primary cultures were grown overnight in LB medium, diluted 1:100 into fresh medium, and grown to mid–late exponential phase (OD₆₀₀ ≈ 2.0). Cells were harvested and washed three times with PBS. Bacterial suspensions were divided into treatment groups: untreated control, acetate (20 mM), GTTR (gentamicin–Texas Red conjugate; 8 μg mL⁻¹), and a combination of acetate and GTTR. Cultures were incubated at 37 °C for 45 min under shaking conditions. Following incubation, cells were harvested, washed twice with 1× PBS to remove excess dye, and fixed in 2% formaldehyde. Fixed cells were diluted in PBS and analyzed using a BD FACSlyric. The Texas Red fluorophore was excited at 488 nm, and fluorescence emission was detected in the PE-Texas Red channel. Fluorescence intensity was used as a proxy for intracellular aminoglycoside.

**Metabolic assays**

Primary cultures were grown overnight in LB medium, diluted 1:100 into fresh medium, and grown to mid–late exponential phase (OD₆₀₀ ≈ 2.0). Cells were harvested and washed three times with PBS. Bacterial suspensions were divided into treatment groups: untreated control, tobramycin (16 μg mL⁻¹), acetate (20 mM), and a combination of acetate and tobramycin. Cultures were incubated at 37 °C for 4 h. Metabolic activity was assessed using a resazurin reduction assay. Briefly, 200 μL of treated bacterial suspensions were transferred to a 96-well plate, and resazurin was added to a final concentration of 5 μg mL⁻¹. Plates were incubated in the dark for 1 h, and absorbance was measured at 570 nm using a Synergy H1 microplate reader. Intracellular ATP levels were quantified using the BacTiter-Glo Microbial Cell Viability Assay according to the manufacturer’s instructions. Briefly, 50 μL of each sample was mixed with 50 μL of BacTiter-Glo reagent in a white 96-well plate. Luminescence was measured using a Synergy H1 microplate reader. Background ATP levels from spent medium were subtracted from each sample, and 1 μM ATP was used as a positive control.

**Reactive oxygen species (ROS) measurement**.

The detailed methodology is provided in the methods section of Supplementary Information. Primary cultures were grown overnight in LB medium, diluted 1:100 into fresh medium, and grown to mid–late exponential phase (OD₆₀₀ ≈ 2.0). Cells were harvested and washed three times with PBS. Bacterial suspensions were divided into treatment groups: untreated control, tobramycin (16 μg mL⁻¹), acetate (20 mM), acetate plus tobramycin, and acetate plus tobramycin in the presence of the ROS quencher ascorbic acid (250 μg mL⁻¹). Cultures were incubated at 37 °C for 4 h. Following treatment, cells were incubated with 2′,7′-dichlorofluorescin diacetate (DCFH-DA; 5 μM) for 30 min. Cells were then washed and resuspended in 1× PBS. Fluorescence was measured in black 96-well plates at excitation/emission wavelengths of 485/528 nm. For flow cytometry analysis, treated cells were processed similarly and analyzed using a BD FACSLyric. Fluorescence was detected in the FITC channel (488 nm excitation, ~530 nm emission), and signal intensity was used as a measure of intracellular ROS levels.

**Generation of clean knockout strains of the sodC gene**

The deletion mutant of the sodC gene was created in *A. baumannii* 17978 VU using a homologous-recombination based recombineering technique. Briefly, a construct carrying an apramycin cassette (amplified from pMDIAI and having FRT sites on both sides) flanking between 500 bp upstream and 500 bp downstream of the gene of interest was cloned into a pUC18 vector (used as a cloning vector). A PCR product was amplified from the construct using a 125 bp upstream forward primer and a 125 bp downstream reverse primer of the gene of interest. Around 5 µg of the concentrated PCR gel-purified product was transformed into A. baumannii electrocompetent cells harboring pAT02 (which contains Rec Ab system) under IPTG induction (5 mM) and plated on LB-agar containing apramycin (30 µg/mL). The transformants were further passaged on LB-agar containing an increasing concentration of apramycin (30 µg/mL). The recombinants were further confirmed by PCR using primers located outside the regions of homology (i.e., 500 bp upstream forward primer and 500 bp downstream reverse primer of the gene of interest) . Following PCR confirmation and curing of pAT02, a clean knockout was created by transforming pAT03 (which contains the FLP recombinase system) under IPTG induction (2 mM). A loss of apramycin resistance confirmed the clean knockout and the losing apramycin-FRT was further confirmed by PCR using apramycin forward and reverse primers.

**Supplementary Figures:**

**Supplementary Fig 1: Metabolic alterations and histopathological changes in high-fat diet–fed mice.
a,** Quantification of serum and liver triglyceride levels in lean and obese mice at baseline uninfected conditions. Total triglyceride concentrations were measured using a ELISA kit according to the manufacturer’s instructions. Data are presented as ng l⁻¹. Each dot represents an individual animal. Data are shown as mean ± s.d; n = 6 animals per group. Significance determined by one-way ANOVA followed by Tukey’s multiple comparison test (*P < 0.01; **P < 0.001; ***P < 0.003; ****P <0.0001; ns, non-significant). **b,** Total cholesterol levels measured in liver and serum from lean and obese mice at baseline. Total cholesterol quantification was performed using ELISA kit following the manufacturer’s protocol. Data are presented as ng l⁻¹. Each dot represents an individual animal. Data are shown as mean ± s.d.; (n = 6 animals per group). Statistical analysis was performed using one-way ANOVA with multiple comparison testing **c–f,** Representative H&E-stained lung sections from lean and diet-induced obese mice. **c**, Uninfected lean lungs showing preserved pulmonary architecture, with patent bronchioles (black stars) and well-defined alveolar spaces (red arrows). **d**, Infected lean lungs showing inflammatory cellular accumulation and congestion around the bronchiolar and alveolar regions, while overall alveolar architecture remains relatively preserved. **e**, Uninfected obese lungs showing increased baseline tissue congestion and inflammatory changes compared with lean lungs, with bronchioles containing intraluminal cellular material (black star). **f**, Infected obese lungs showing more pronounced pulmonary inflammation and tissue congestion, with increased cellular accumulation around the airways and alveolar regions and greater disruption of normal lung architecture. Representative images were acquired by bright-field microscopy at 400× magnification.

**Supplementary Fig 2: Temporal dynamics of systemic inflammatory responses in lean and obese mice following infection.**
**a,** Schematic representation of the experimental design. Lean and high-fat diet–induced obese C57BL/6 mice were subjected to systemic infection (day 0), followed by longitudinal blood sampling at days 0 (prior to infection), 1, 7, and 14 post-infection for downstream analyses. **b–e,** Time-resolved quantification of circulating cytokines in lean and obese mice following infection. Serum cytokine levels were measured at baseline (pre-infection) at day 0, day 1, day 7, and day 14 post-infection using enzyme-linked immunosorbent assay (ELISA) according to the manufacturer’s instructions. Cytokine concentrations are expressed as pg ml⁻¹. **b,** TNF-α; **c,** IL-6; **d,** IFN-γ; **e,** IL-1β. Data are presented as mean ± s.d., with each point representing n = 6 animals per group. Statistical significance was determined using two-way analysis of variance (ANOVA) with Tukey’s multiple comparison test to assess differences between lean and obese groups across time points. P values are indicated in the plots.

**Supplementary Fig 3: Obesity-associated alterations in gene expression of organs homogenates following infection.
a**, Schematic representation of the experimental workflow. Lean and high-fat diet (HFD)–induced obese C57BL/6 mice were subjected to infection (day 0), followed by organ harvesting at day 1 post-infection for gene expression analysis. **b–i,** Relative gene expression profiles in lung and kidney tissues from lean and obese mice under uninfected (UI) and infected (IV) conditions. Total RNA was extracted from harvested lung tissues, reverse transcribed into cDNA, and quantitative real-time PCR (qRT–PCR) was performed using gene-specific primers. Gene expression levels were normalized to housekeeping controls and expressed as fold change relative to lean uninfected controls at day 0. **b,** Tlr4; **c,** Il6; **d,** Ifn-γ; **e,** Tnf-α; **f,** IL-1β; **g** Cxcl3; **h,** Cxcl2; **i,** Ffar2 **j,** Ffar3. Each dot represents mean of three individual biological replicate. Data are presented as mean ± s.d. Significance determined by one-way ANOVA followed by Tukey’s multiple comparison test (*P < 0.01; **P < 0.001; ***P < 0.003; ****P <0.0001; ns, non-significant). All experiments were independently repeated twice with similar results.

**Supplementary Fig 4: High-fat diet–associated gut microbiota dysbiosis and partial restoration by acetate supplementation**.
**a–c**, Alpha diversity metrics of gut microbiota in lean and obese mice. Fecal samples were collected and subjected to 16S rRNA sequencing, followed by bioinformatic analysis using standard pipelines. **a,** Species evenness (Shannon index), **b,** species richness (Chao1 index), and **c**, species diversity (Simpson index). Each dot represents an individual biological replicate (n = 3 mice per group). Data are presented as mean ± s.d. Significance determined by one-way ANOVA followed by Tukey’s multiple comparison test (*P < 0.01; **P < 0.001; ***P < 0.003; ****P <0.0001; ns, non-significant). **d,** Heat map showing relative abundance of dominant bacterial phyla across experimental groups, including lean control mice, obese mice, and obese mice treated with acetate (DW for 30 days). Taxonomic classification was performed based on 16S rRNA sequencing data, and relative abundances were normalized and scaled (z-score) across samples. Hierarchical clustering was applied to both taxa and samples to visualize compositional differences. All experiments were independently performed with comparable results.

**Supplementary Fig 5: High-fat diet reduces antibiotic efficacy in systemic infection models.**
**a**, Schematic representation of the experimental design. C57BL/6 mice were maintained on either a standard diet or high-fat diet (HFD) for 70 days, followed by systemic infection. Antibiotic treatment was administered 6 h post-infection, and organ samples were collected 24 h post-infection for bacterial burden analysis. **b–d,** In vivo bactericidal efficacy of different classes of antibiotics in lean and obese mice. Mice were infected with a non-lethal bacterial inoculum and treated with antibiotics, including tobramycin (TOB; 10 mg kg⁻¹), polymyxin B (POL B; 5 mg kg⁻¹), meropenem (MEM; 100 mg kg⁻¹), and rifampicin (RIF; 25 mg kg⁻¹), administered at 6 h post-infection. Bacterial loads were quantified in **b,** lungs, **c**, liver, and **d**, kidneys. Organs were aseptically harvested, homogenized in sterile phosphate-buffered saline, serially diluted, and plated for colony-forming unit (c.f.u.) enumeration. Data are presented as log₁₀ reduction in c.f.u. relative to untreated controls. **e,** Serum concentrations of tobramycin measured 30 min post intraperitoneal administration (i.p.) in lean and obese mice. Drug levels were quantified using ELISA kit according to manufacturer’s protocol. Data are presented as pg ml⁻¹. Each dot represents an individual biological replicate (n = 6 mice per group). Data are shown as mean ± s.d. Statistical significance was determined using two-way analysis of variance (ANOVA) with Tukey’s multiple comparison test **(b–d)** or unpaired two-tailed Student’s t-test **(e)**. (*P < 0.01; **P < 0.001; ***P < 0.003; ****P <0.0001; ns, non-significant).

**Supplementary Fig 6: Acetate supplementation restores gut microbiota composition, systemic SCFA levels, and FFAR2 signaling in obese mice following fecal microbiota transplantation (FMT).**

**(a)** Schematic representation of the experimental design. Mice were first treated with an antibiotic cocktail for 7 days to deplete the native gut microbiota. Animals were then divided into three groups based on donor fecal microbiota: (i) lean control FMT, (ii) obese FMT, and (iii) obese FMT with acetate supplementation. Fecal slurries were orally administered daily for 14 days. During this period, mice received normal chow and water, with acetate provided to the designated donor group. At the end of the treatment period, animals were subjected to experimental procedures and sample collection**. (b–d)** Relative abundance of major gut bacterial taxa following FMT, quantified by 16S rRNA gene levels by deltaCt method and expressed as relative abundance (mean ± SEM). **(b)** Bacteroides abundance was significantly reduced in mice receiving obese FMT compared to lean controls, and this reduction was partially restored upon acetate supplementation. **(c)** Firmicutes abundance was significantly elevated in obese FMT recipients relative to lean controls, with acetate treatment normalizing this increase **(d)** Akkermansia abundance was markedly decreased in obese FMT mice and restored upon acetate supplementation, indicating recovery of beneficial mucin-degrading bacteria. **(e–f)** Quantification of acetate levels in systemic and tissue compartments using targeted metabolite assays. **(e)** Serum acetate concentrations (mM) were significantly reduced in obese FMT mice compared to lean controls and were restored upon acetate supplementation **(f)** Lung acetate levels (mM) showed a similar trend, with reduced levels in obese FMT mice and significant recovery following acetate treatment **(g)** Measurement of FFAR2 levels in lung tissue (ng/L), determined by ELISA. Obese FMT resulted in a marked downregulation of FFAR2 expression compared to lean controls, while acetate supplementation significantly restored FFAR2 levels, indicating recovery of acetate-mediated immune signaling. Each dot represents an individual biological replicate. Significance determined by one-way ANOVA followed by Tukey’s multiple comparison test (*P < 0.01; **P < 0.001; ***P < 0.003; ****P <0.0001; ns, non-significant).

**Supplementary Fig 7: Acetate supplementation restores antibiotic efficacy and reduces bacterial burden in multiple organs following obese microbiota transplantation.**

**(a–c)** Bacterial burden (Log₁₀ CFU/g tissue) in lungs **(a)**, liver **(b)**, and kidney **(c)** of mice receiving fecal microbiota transplantation (FMT) from normal diet (ND), high-fat diet (HFD), or acetate-treated obese donors. Following FMT, mice were subjected to infection and either left untreated or treated with Tobramycin (10mg/kg body weight). Bacterial loads were quantified by plating tissue homogenates on appropriate agar media and expressed as colony-forming units (CFU) per gram of tissue (log₁₀-transformed). Significance determined by one-way ANOVA followed by Tukey’s multiple comparison test (n=5 animals per group) (*P < 0.01; **P < 0.001; ***P < 0.003; ****P <0.0001; ns, non-significant).

**Supplementary Fig 8:** **a-c**, **Acetate potentiates tobramycin-mediated bacterial clearance in obese mice infected with PAO1; and increase in-vitro efficacy of tobramycin against other Gram negative pathogens.** Diet-induced obese mice were infected with *P. aeruginosa* PAO1 and subsequently treated with vehicle (untreated), tobramycin alone (10 mg/kg body weight), acetate alone (500 mg/kg body weight), or a combination of tobramycin and acetate. Bacterial burdens were quantified in the **(a)** lungs, **(b)** liver, and **(c)** kidneys by plating tissue homogenates and are expressed as log₁₀ CFU per gram of tissue. Combination treatment (tobramycin + acetate) resulted in a significant reduction in bacterial load across all organs compared to monotherapy groups. Each data point represents an individual mouse, and horizontal bars indicate mean ± [SD/SEM]. **d–f,** Time–kill kinetics of Gram-negative bacterial pathogens under different treatment conditions. Bacterial cultures were treated with acetate (20 mM), tobramycin (16 μg ml⁻¹), or a combination of acetate and tobramycin, and viable counts were determined at the indicated time points by plating serial dilutions on agar for colony-forming unit (c.f.u.) enumeration. Data are presented as log₁₀ c.f.u. ml⁻¹. The dashed line indicates the limit of detection (LOD). **a,** Escherichia coli ATCC 25922; **b,** P. aeruginosa (PA01); **c,** K. pneumonia ATCC 700698 (Each condition includes untreated controls and single-agent treatments for comparison. Data represent mean ± s.d. from independent experiments (n = 3). Significance determined by one-way ANOVA followed by Tukey’s multiple comparison test (*P < 0.01; **P < 0.001; ***P < 0.003; ****P <0.0001; ns, non-significant).

**Supplementary Fig 9:** **Acetate potentiates tobramycin activity through metabolic disruption and reactive oxygen species (ROS) generation in A. baumannii 17978 VU.** **a–c,** Metabolic and oxidative changes in bacteria following treatment. Bacterial cultures were treated with acetate (20mM), tobramycin (8 μg ml⁻¹), or a combination of acetate and tobramycin. **a,** Metabolic activity was assessed using a resazurin based colorimetric assay and measured as absorbance at 570 nm. Data are presented as mean ± s.d. from independent experiments (n = 3) **b,** Intracellular ATP levels were determined using a luminescence-based ATP assay. Data are presented as mean ± s.d. from independent experiments (n = 3) **c,** Intracellular ROS generation was measured using a ROS-sensitive fluorescent dye DCFH-DA, and fluorescence intensity was normalized across conditions. Data are presented as susing one-way analysis of variance (ANOVA) with multiple comparison testing. P values are indicated in the plots. **d,** Flow cytometry analysis of intracellular ROS levels under indicated treatment conditions. Bacterial cells were stained with a ROS-sensitive fluorescent probe DCFH-DA, and analyzed by flow cytometry (FITC-A channel). Representative histograms show shifts in fluorescence intensity corresponding to ROS accumulation.; VC, vitamin C (ROS scavenger). **e,** Survival of wild-type and oxidative stress–deficient bacterial strains following treatment with acetate and tobramycin. Mutant strains lacking ROS-detoxifying enzymes (ΔsodB:Tn and ΔsodC) were compared to the wild-type strain (17978 VU). Bacterial survival is presented as log₁₀ (survival fraction). Complementation of sodB and sodC in pWkBAD plasmid reversed the effect of the combination. Data are shown as mean ± s.d. (n = 3). Statistical significance was determined using one-way ANOVA with multiple comparison testing. Significance determined by one-way ANOVA followed by Tukey’s multiple comparison test (*P < 0.01; **P < 0.001; ***P < 0.003; ****P <0.0001; ns, non-significant). **f,** Effect of ROS quenching on bactericidal activity. Bacterial cultures were treated with acetate (20mM) and/or tobramycin in the presence or absence of ROS scavengers (vitamin C- 250 µg/ml). Viable counts were determined over time by plating for colony-forming unit (c.f.u.) enumeration. Data are presented as log₁₀ c.f.u. ml⁻¹. The dashed line indicates the limit of detection (LOD).

**Table S1: List of strains used in this study**

| **Strain:** |  |
| --- | --- |
| *A. baumannii* ATCC 17978 VU | Purchased from ATCC |
| *K. pneumoniae* ATCC 700698 | Purchased from ATCC |
| *P. aeruginosa* PAO1 | Purchased from ATCC |
| *E. coli* 25922 | Purchased from ATCC |
| *Acinetobacter baumannii* 17978 VU Δ*sodB:Tn* | Gift from Koch Institute |
| *Acinetobacter baumannii* 17978 VU Δ*sodB:Tn/psodB* | This study |
| *Acinetobacter baumannii* 17978 VU ΔsodC | This study |
| *Acinetobacter baumannii* 17978 VU ΔsodC:sodC | This study |

**Table S2: MIC values of tobramycin and gentamycin against various Gram negative strains.**

| **Strain No:** | **Tobramycin (μg/ml)** | **Gentamycin (μg/ml)** |
| --- | --- | --- |
| *A. baumannii* ATCC 17978 VU | 2 | 2 |
| *K. pneumoniae* ATCC 700698 | 4 | 16 |
| *P. aeruginosa* PAO1 | 2 | 16 |
| *E. coli* 25922 | 4 | 16 |

**Table S3**. List of the bacterial plasmids that were used in the current study.

| Sl. No. | Plasmid | Characteristics | Source/references |
| --- | --- | --- | --- |
| 1 | pUC18 | Cloning vector in *E. coli*; amp | Thermo Scientific, USA |
| 2 | pMDIAI | Template for apramycin cassette with FRT sites on both sides | Addgene |
| 3 | pAT02 | Plasmid expressing *A. baumannii* RecAB, AmpR | Dr. Bryan Davies, University of Texas, Austin, Texas, USA |
| 4 | pAT03 | Plasmid expressing FLP recombinase enzyme (flippase) for expression in *A. baumannii*, AmpR | Dr. Bryan Davies, University of Texas, Austin, Texas, USA |
| 5 | pWkBAD30 | Plasmid (KanR) used for complementation of *sodB* and *sodC* gene in *A. baumannii* 17978 VU | Lab stock |

**Table S4: List of primers used in the study (for qPCR and cloning).**

|  | Oligonucleotide name | Primers | Sequence |
| --- | --- | --- | --- |
| 1 | FFAR3_RT | Forward | GTCTGTTGGTTCCTGGCATC |
| 2 | FFAR3_RT | Reverse | CCTGGCTGTAGGTTGCATTT |
| 3 | IL-1β_RT | Forward | GGCTGGACTGTTTCTAATGC |
| 4 | IL-1β_RT | Reverse | ATGGTTTCTTGTGACCCTGA |
| 5 | IL-6_RT | Forward | GAAACCGCTATGAAGTTCCTCTCTG |
| 6 | IL-6_RT | Reverse | TGTTGGGAGTGGTATCCTCTGTGA |
| 7 | TNF-α_RT | Forward | GGGTGTTCATCCATTCTC |
| 8 | TNF-α_RT | Reverse | GGAAAGCCCATTTGAGT |
| 9 | IL-6_RT | Forward | ATGGCTGGGATTCACCTCAA |
| 10 | IL-8_RT | Reverse | AAGCCTCGCGACCATTCTT |
| 11 | Cxcl2_RT | Forward | GGACCCGGAGTGCCATAGAA |
| 12 | Cxcl2_RT | Reverse | TCCAACAGATATGCCTGCTCAT |
| 13 | Cxcl3_RT | Forward | TTTGAGACCATCCAGAGCTTG |
| 14 | Cxcl3_RT | Reverse | CCTTGAGAGTGGCTATGACTTC |
| 15 | Tlr4_RT | Forward | CCAATTTTTCAGAACTTCAGTGGC |
| 16 | Tlr4_RT | Reverse | AGAGGTGGTGTAAGCCATGC |
| 17 | Ffar2_RT | Forward | CCCGGTTTTGGTAGGTTCCAC |
| 18 | Ffar2_RT | Reverse | AGGCCGTGAGGATCAAGGAAC |
| 19 | Universal16S_RT | Forward | TCCTACGGGAGGCAGCAGT |
| 20 | Universal16S_RT | Reverse | GGACTACCAGGGTATCTAATCCTGTT |
| 21 | Akkermansia_RT | Forward | CAGCACGTGAAGGTGGGGAC |
| 22 | Akkermansia_RT | Reverse | CCTTGCGGTTGGCTTCAGAT |
| 23 | Bacteroides_RT | Forward | GAGAGGAAGGTCCCCCAC |
| 24 | Bacteroides_RT | Reverse | CGCTACTTGGCTGGTTCAG |
| 25 | Firmicutes_RT | Forward | GGAGYATGTGGTTTAATTCGAAGCA |
| 26 | Firmicutes_RT | Reverse | AGCTGACGACAACCATGCAC |
| 27 | Bac16S_RT | Forward | AGAGTTTGATCCTGGCTCAG |
| 28 | Bac16S_RT | Reverse | AAGGAGGTGWTCCARCC |
| 29 | Universal_16S_RT | Forward | AAACTCAAAKGAATTGACGG |
| 30 | Universal_16S_RT | Reverse | CTCACRRCACGAGCTGAC |
| 31 | SodC_US500 | Forward | CCCAAGCTTTGCAACGTTCTTATTAGC |
| 32 | SodC _US500 | Reverse | CGCGGATCCTATAGATATCCTTATAACTATAAATC |
| 33 | SodC_DS500 | Forward | GGCGGTACCATGCTATTTTTAAATTGTGG |
| 34 | SodC_DS500 | Reverse | CGGAATTCGCTTTAAAACGATTGAATAGTTG |
| 35 | SodC_US125 | Forward | GATGATGATTTACCGCCATC |
| 36 | SodC_DS125 | Reverse | GAGGGTACGTGACAATTGG |
| 37 | Apr_BamH1 | Forward | ATCAGGATCCGTCGACCTGCAGTTC |
| 38 | Apr_Kpn1 | Reverse | ATGGTACCGTGTAGGCTGGAGCTGCTTC |
| 39 | sodB_EcoRI | Forward | CCGGAATTCATGACAACCATTACTTTACC |
| 40 | sodB_KpnI | Reverse | CGGGGTACCTTATTTCTCTACACCAGCTGG |
| 41 | sodC_EcoRI (native with signal peptide) | Forward | GATTACGAATTCATGCCAGTATTTAATAAAATTGGTGCAGTATGTGCACTGACAGCTT |
| 42 | sodC_KpnI_RP | Reverse | ATCGGTACCTTATTTGATTACACCACATGC |

**References:**

1.	Ross AL, Tharp JL, Hobbs GR, McKnight R, Cumpston A. 2013. Evaluation of extended interval dosing aminoglycosides in the morbidly obese population. Adv Pharmacol Sci 2013:194389.

2.	Wang P, Zhang Q, Feng M, Sun T, Yang J, Zhang X. 2021. Population Pharmacokinetics of Polymyxin B in Obese Patients for Resistant Gram-Negative Infections. Front Pharmacol 12:754844.

3.	Ana C-B, Iria V-R, Beatriz M, Cristina M-G, Irene Z-F, Teresa R-J, Anxo F-F. 2024. Updated antimicrobial dosing recommendations for obese patients. Antimicrob Agents Chemother 68:e01719-23.

4.	Meng L, Mui E, Ha DR, Stave C, Deresinski SC, Holubar M. 2023. Comprehensive guidance for antibiotic dosing in obese adults: 2022 update. Pharmacother J Hum Pharmacol Drug Ther 43:226–246.

5.	Ray AK, Bhowmik S, Saini S, Hussain A, Bakht P, Pandey S, Pathania R. 2025. Manganese-dependent iron-superoxide dismutase drives <em>Acinetobacter baumannii</em> fitness during oxidative stress. J Biol Chem 301.
